# ﻿A decade of amphibian studies (Animalia, Amphibia) at Sekayu lowland forest, Hulu Terengganu, Peninsular Malaysia

**DOI:** 10.3897/zookeys.1157.95873

**Published:** 2023-03-31

**Authors:** Baizul Hafsyam Badli-Sham, Muhamad Fatihah Syafiq, Mohd Shahrizan Azrul Aziz, Natrah Rafiqah Mohd Jalil, Muhammad Taufik Awang, Muhammad Nouril Ammin Othman, Anis Azira Abdul Aziz, Khunirah Dzu, Nurul Asyikin Abdol Wahab, Nor Liyana Jamil, Murni Azima Ismail, Wan Ahmad Aidil Wan Azman, Ooi Xin Wei, Nur Ain Nabilah Jamaha, Mohamad Aqmal-Naser, Muhammad Fahmi-Ahmad, Noor Shahirah-Ibrahim, Syed Ahmad Rizal, Daicus M. Belabut, Chan Kin Onn, Evan Seng Huat Quah, Larry Lee Grismer, Amirrudin B. Ahmad

**Affiliations:** 1 Biodiversity and Ecology Research Group, Faculty of Science and Marine Environment, Universiti Malaysia Terengganu, 21030 Kuala Nerus, Terengganu, Malaysia; 2 Institute of Tropical Biodiversity and Sustainable Development, Universiti Malaysia Terengganu, 21030 Kuala Nerus, Terengganu, Malaysia; 3 Academy of Science Malaysia, 902-4, Jalam Tun Ismail, 50480 Kuala Lumpur, Malaysia; 4 Forestry Biotechnology Division, Forest Research Institute Malaysia, 52109 Kepong, Selangor, Malaysia; 5 Institute of Biological Sciences, Faculty of Science, University of Malaya, 50603 Kuala Lumpur, Malaysia; 6 Lee Kong Chian Natural History Museum, National University of Singapore, 2 Conservatory Drive, 117377 Singapore, Singapore; 7 Institute for Tropical Biology and Conservation, Universiti Malaysia Sabah, 88400 Kota Kinabalu, Sabah, Malaysia; 8 Department of Biology, La Sierra University, 4500 Riverwalk Parkway, Riverside, California, 92515-8247 USA

**Keywords:** Biodiversity conservation, herpetofauna, lowland forest, Malaysia, protected areas

## Abstract

Amphibians of Sekayu lowland forest have been studied more than a decade, with discoveries of new records of species showing no sign of abating between the years 2003 to 2020, indicating the remarkably rich diversity of anurans in this forest. Despite ceaseless anthropogenic activities in this area, this study successfully recorded 52 species of amphibians from 32 genera in the lowland forest of Sekayu. The species composition consisted of a single species from the family Ichthyophiidae and 51 species of anurans of 31 genera and six families. The number of species recorded has steadily increased especially during more recent surveys from 2015 to 2020. This study augments the total number of amphibian species recorded from Hulu Terengganu by ten additional species, increasing the total to 70 species for the district.

## ﻿Introduction

The earliest herpetological surveys conducted by Dring (1979) at Gunung Lawit, Hulu Terengganu reported 77 species, of which 44 were amphibians. The survey also discovered two new species of geckos, *Cyrtodactyluselok* and *Cnemaspisargus*. Until the 1990s, limited herpetological studies had been conducted, such as the surveys on reptiles in Bukit Labohan in Ma’ Daerah by Davison (1993), which enlisted at least ten reptile species, and the surveys on the freshwater turtle trade conducted by Sharma (1999) in several districts in Terengganu. Surveys from 2000 to 2010 consist of amphibian studies by Norhayati et al. (2006) in Pasir Raja Forest Reserve and a follow-up survey on a reptile in Bukit Labohan by Sharma et al. (2007) with photograph records of amphibians. In 2003 and 2008, an Environmental Impact Assessment (EIA) on herpetofauna was done by Tenaga Nasional Berhad Research (TNBR) in Tembat Forest Reserve before the construction of two dams began in this areas (TNBR 2003, 2007). Two new species were made within this period, namely *Cnemaspisperhentianensis* on Pulau Perhentian Besar (Grismer and Chan 2008) and *Cyrtodactylusleegrismeri* on Pulau Tenggol (Chan and Norhayati 2010).

From 2011 to the present, extensive efforts have been made to document the herpetofauna diversity in Terengganu, including lowland to upland areas (Gunung Gagau: Hamidi 2013; Gunung Tebu and Lata Belatan recreational forest: Muin et al. 2014; the base of Gunung Lawit, Gunung Tebu, adjacent lowland forests of Lata Tembakah and Lata Belatan: Sumarli et al. 2015; Tasik Kenyir: Zakaria et al. 2019; Komaruddin et al. 2020), wetlands (Setiu: Tamblyn et al. 2006; Zahidin et al. 2017), urbanised areas (UMT Campus: Badli-Sham et al. 2019) and archipelagic islands (Perhentian archipelago, Pulau Redang, and Pulau Tenggol: Grismer et al. 2011; Pulau Bidong: Zakaria et al. 2015; Fatihah-Syafiq et al. 2020). Still, continuous surveys were carried out in Tembat Forest Reserve to monitor the herpetofauna communities during the construction of hydroelectric dams (Chan 2011; Norhayati et al. 2011; Ummi 2013; Nur Amalina et al. 2017, 2020, 2021). Surprisingly, more new species were discovered during this period, such as *Lipiniasekayuensis* (Grismer et al. 2014, 2016), *Tytthoscincuskeciktuek* and *T.monticulous* (Grismer et al. 2018) and *Rentapiaflavomaculata* (Chan et al. 2020a), and the discovery of *Philautusdavidlabangi* (Quah et al. 2021) in Tasik Kenyir which is a new record for Peninsular Malaysia. Besides, many localities in Terengganu are still under ongoing surveys by a herpetology team from Biodiversity and Ecology Research Group (BERes), Universiti Malaysia Terengganu, particularly in the Hulu Terengganu District.

As part of the Hulu Terengganu Forest Reserve that is adorned with beautiful streams and intact forests, Sekayu lowland forest (SLF) has become the most popular picnic spot in Terengganu amongst locals and tourists alike with nearly 203,000 visitors reported in the year 2010 (Bhuiyan et al. 2011). Accommodated with sufficient facilities, this area has become the most conducive place for recreational and ecotourism activities. SLF is known to house a diverse range of organisms, such as butterflies, aquatic invertebrates (Wahizatul et al. 2011; Wahizatul and Geok 2016) and dragonflies (Wahizatul et al. 2006; Choong et al. 2013), reptiles (Zakaria et al. 2019), fishes (Kottelat et al. 1992; Tan and Ng 2005), and also diverse families of trees (Jarina et al. 2007). Several species of reptiles have been described from this area such as skinks, *Lipiniasekayuensis* (Grismer et al. 2014, 2016), *Tytthoscincuskeciktuek* and *T.monticolus* (Grismer et al. 2018), plus a new genus and species of terrestrial crab, *Gempalabilobata* (Ng and Ahmad 2016), and *Johoramichaeli* (Ng 2020). Recent fieldwork in SLF has contributed additional locality records for several recently described species in Peninsular Malaysia such as the skink *Sphenomorphussungaicolus* (Sumarli et al. 2016) and *Rentapiaflavomaculata* (Chan et al. 2020a).

Furthermore, the actual diversity of amphibians in SLF remains uncertain as the checklists were not properly reviewed and updated in the latest taxonomy, may contain several erroneous records or misidentifications of specimens, and the existing areas have not been exhaustively surveyed. The objectives of this paper are to: (1) properly compile and update the information from previous and recent fieldwork to produce a comprehensive checklist of amphibians, (2) to examine the previously and recently collected specimens in SLF for accurate species accounts, and (3) to assess the trend of long-term surveys conducted on amphibians throughout the past decade in SLF. In addition, this paper also provides a compiled checklist based on published records on the amphibian fauna in Hulu Terengganu District (Dring 1979; Norhayati et al. 2011; Hamidi 2013; Sumarli et al. 2015; Nur Amalina et al. 2017) to report the current diversity of amphibians in this area.

## ﻿Materials and methods

### ﻿Study area

**Figure 1.**
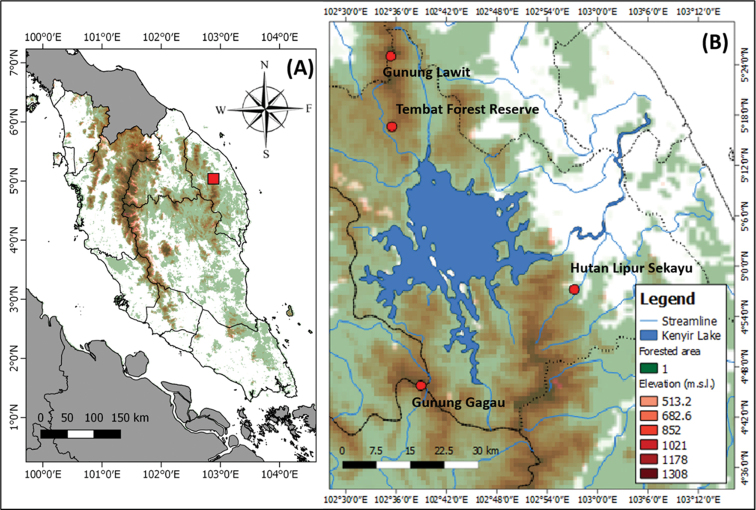
Map **A** shows the location of Sekayu lowland forest (SLF) indicated by red box in Peninsular Malaysia. The detailed location of SLF and several localities with published checklist on amphibians in Hulu Terengganu were displayed on Map **B** and indicated by the red circles for respective localities.

Sekayu lowland forest (**SLF**) is located within the Hulu Terengganu Forest Reserve (Annex) near Kuala Berang to the east, and Taman Negara (= National Park) to the west and south-west (Fig. 1), with a total area of 30 ha. This area consists of Sekayu Recreational Forest (**SRF**) that is covered by tall and old growth lowland dipterocarp trees, cascading waterfalls drained by a pristine stream from the Peres River, and Sekayu Agricultural Park (**SAP**) which is an enormous agricultural area of 85 ha that is also open for recreational activities, and is drained by the Bubu River (Wahizatul et al. 2011). This area receives heavy rainfall from the northeast monsoon that typically occurs between October and March of each year (Khan et al. 2014).

### ﻿Descriptions of study sites

In SLF, fieldworks were conducted at various locations within SRF and SAP. The Sekayu Recreational Forest area (SRF) (entrance at 4°58'01.1"N, 102°57'35.4"E) contains substantial landscape changes to accommodate facilities for Forestry staff and visitors, such as huts, toilets, changing rooms, chalets, and camping sites. SRF is drained by a stream channel of the Peres River, which remains the main attraction for this area for recreational activities such as picnics and camping (Bhuiyan et al. 2011). The trekking route at SRF is also used as the main hiking route to Gunung Gajah Terom. Minor modifications were observed at certain parts of the Peres River, such as the construction of a dam to create shallow pools and suspension bridges that provided access to the forest within the SRF. However, a large portion of trees families and many others vegetations remain intact and well preserved (Jarina et al. 2007; Rafаai 2007; Nor 2007). In addition, many microhabitats such as rock pools, riparian vegetation, and small streams located beyond the recreational area are untouched. Much of the area in the recreational zone consists of a mixture of natural forest vegetation and garden plants, along with artificial drainage, especially at the Herbal Park. The surveys were conducted at six sampling sites within the SRF, which included the upper stream area and a tributary of the Peres River, recreational zone (vicinity of the car park, chalet, Forestry office, and trekking route), Orchid Garden, Herbal Park, and camping site.

Sekayu Agricultural Park (SAP) (entry point at 4°58'01.8"N, 102°57'28.4"E) is comprised of mostly agricultural lands growing various species of fruits such as *Lansiumdomesticum*, *Nepheliumlappaceum*, *Graciamangostana*, *Duriozibethinus*, and garden plants. Most of the landscape of this area is significantly altered and surrounded by the forest edge and drained by a stream channel of the Bubu River (Wahizatul et al. 2011). There is an upstream area of the Bubu River (4°58'20.12"N, 102°57'26.86"E) that was predominantly covered by secondary forest as in SRF, along with beautiful cascading waterfalls, an untouched small stream, and an abundance of granitic boulders along the upstream area. Similar to SRF, this area was occasionally visited by locals and has undergone moderate landscape modifications with the construction of recreational facilities such as a suspension bridge, Herbal Park and swimming pool. Half a kilometre from this area, buildings such as rest houses, dormitory and camping sites are provided. However, many of these buildings have been abandoned due to lack of maintenance. The upstream area of the Bubu River was frequently observed to have severe sediment loads, especially in the monsoon season which is assumed to have resulted from logging activities in nearby areas. The surveys were conducted in five sampling sites within the SAP, such as the upper stream area and a tributary of Bubu River, forest trail, recreational zone (area of rest houses, dormitory and camping site), and Herbal Park.

### ﻿Data collection

Surveys were conducted using standard methods of visual encounter surveys (**VES**) and acoustic surveys, together with drift-fenced pitfall traps in SLF. VES is a time-constrained technique that is frequently employed in herpetological surveys, in which the observers walk along a in standardised route at a standard pace to visually search the entire area for amphibians (Crump and Scott 1994; Doan 2003). Any calling heard during the surveys were identified by the help of experts and/or calls were referenced at AmphibiaWeb (2021) (https://amphibiaweb.org). Five sets of drift-fenced pitfall traps (each set comprised of three pitfall traps and two 2.5-m long aluminium sheets) were set up for each of the small feeder streams of the Peres River (PR1: 4°57'43.15"N, 102°57'12.16"E; PR2: 4°57'42.43"N, 102°57'11.57"E; PR3: 4°57'42.11"N, 102°57'10.24"E; PR4: 4°57'40.03"N, 102°57'9.04"E; and PR5: 4°57'39.73"N, 102°57'7.2"E) at SFR and along the hiking trail at SAP (HT1: 4°58'17.7"N, 102°57'21.25"E; HT2: 4°58'17.34"N, 102°57'21.12"E; HT3: 4°58'16.99"N, 102°57'21.17"E; HT4: 4°58'16.53"N, 102°57'21.08"E; and HT5: 4°58'16.28"N, 102°57'21.24"E). Four days and three nights were spent for each survey occasion in (1) October and November 2015, (2) March to May and July to November 2016, (3) September to December 2017, (4) August to December 2018, and (5) July to December 2020.

Additional information of species from SLF was obtained from the compilation of amphibian checklists conducted by previous undergraduate fieldwork from 2003, 2004, 2006, 2013, 2014 and early 2015. Voucher specimens collected from previous and more recent fieldwork were examined to confirm species identifications based on Berry (1975), Brown and Guttman (2002), Harvey et al. (2002), Wood et al. (2008b), Matsui (2006), McLeod and Norhayati (2007), Nishikawa et al. (2012), Chan et al. (2014a, b, 2016, 2018, 2020a–c), Rujirawan et al. (2013), Sumarli et al. (2015), Zug (2015), Matsui et al. (2014, 2017, 2018), Sheridan and Stuart (2018), Davis et al. (2018), Garg et al. (2019), Jiang et al. (2019), and Hong et al. (2021). The latest taxonomic nomenclatural follows the Amphibian Species of the World online database (Frost 2022). Measurement of snout-vent length (**SVL**) were taken using digital callipers. All preserved specimens were designated with the code Universiti Malaysia Terengganu Zoological Collections (**UMTZC**) and deposited in the General Laboratory of Biology, Universiti Malaysia Terengganu. The voucher photographs included in species accounts were designated with the code **UMTZCP** (Universiti Malaysia Terengganu Zoological Collections Photograph).

### ﻿Data analysis

The checklist of amphibians from previous studies (2003–2015) were tabulated and compiled together with the most recent studies (October 2015-December 2020) to obtain the accumulated number of amphibian species that have been recorded in SLF. Species accumulation curve and estimated number of duplicates (species with two samples) and unique species (species represented by one sample) (Colwell and Coddington 1994) of amphibians from SLF was generated from the incidence-based data of species obtained from the year 2003 until 2018 by using EstimateS software 9.0 (Colwell 2013).

## ﻿Results

Fifty-two species of amphibians were recorded in total, consisting of one caecilian from the family Ichthyophiidae and 51 anurans from 31 genera and six families in SLF (Table 1). Thirty-eight species were recorded from previous fieldwork between the years 2003 until early 2015, and 13 additional species were recorded in recent surveys from late 2015 to 2020 in SLF (Table 1). The additional species obtained in the recent surveys were Ichthyophiscf.asplenius, *Ansonialumut*, *Leptophryneborbonica*, *Limnonectesplicatellus*, *L.hascheanus*, *L.utara*, *Occidozygasumatrana*, *O.martensii*, *Kalophrynuspalmatissimus*, *Micrylettadissimulans*, *Pulchranalaterimaculata*, *Polypedatescolletti*, and *Thelodermalicin*. Species nomenclature was updated based on the latest taxonomy, such as *Kaloulalatidisca* (formerly reported as *Kaloulabaleata*) (Chan et al. 2014b), *Amolopsgerutu* (formerly known as *A.larutensis*) (Chan et al. 2018), *Sylviranamalayana* (formerly known as *H.nigrovittata*) (Sheridan and Stuart 2018), *Kurixaluschaseni* (formerly known as *K.appendiculatus*) (Matsui et al. 2018), *Limnonectesdeinodon* (formerly known as *L.laticeps* and *L.khasianus*) (Dehling 2014), *Pulchranasundabarat* (formerly known as *H.picturata*) (Chan et al. 2020c), *Zhangixalusprominanus* and *Z.tunkui* (formerly known as *Rhacophorusprominanus* and *R.tunkui*) (Jiang et al. 2019) and *Rentapiaflavomaculata* (formerly known as *R.hosii*) (Chan et al. 2016; Chan et al. 2020a) were recorded in SLF as well.

**Table 1.** Checklist of amphibians of Sekayu lowland forest, Hulu Terengganu, Terengganu.

No	Family/Species	Year										
		2003	2004	2006	2008	2013	2014	2015	2016	2017	2018	2020
	**Family Ichthyophiidae**											
1	Ichthyophiscf.asplenius	–	–	–	–	–	–	–	–	+	–	–
	**Family Bufonidae**											
2	*Ansonialatiffi*	–	–	–	–	+	+	+	+	–	+	–
3	*Ansonialumut*	–	–	–	–	–	–	–	+	+	+	–
4	*Duttaphrynusbengalensis* (*Duttaphrynus* sp. 1)	–	–	–	+	+	–	–	+	–	+	+
5	*Ingerophrynusparvus*	+	+	+	–	+	+	+	+	+	+	+
6	*Leptophryneborbonica*	–	–	–	–	–	–	–	+	–	–	+
7	*Phrynoidisasper*	+	+	+	+	+	+	+	+	+	+	+
8	*Rentapiaflavomaculata*	–	–	–	–	–	–	+	+	–	–	–
	**Family Dicroglossidae**											
9	*Fejervaryalimnocharis*	+	+	+	+	+	+	+	+	+	+	+
10	*Limnonectesblythii*	+	+	+	+	+	–	+	+	+	+	+
11	*Limnonecteshascheanus*	–	–	–	–	–	–	–	+	+	+	+
12	*Limnonectesdeinodon*	–	–	–	–	–	–	+	+	+	+	+
13	*Limnonectesmalesianus*	+	+	–	–	–	–	+	+	+	+	+
14	*Limnonectesplicatellus*	–	–	–	–	–	–	–	+	+	+	+
15	*Limnonectesutara*	–	–	–	–	–	–	–	–	–	+	–
16	*Occidozygasumatrana*	–	–	–	–	–	–	–	+	–	+	+
17	*Occidozygamartensii*	–	–	–	–	–	–	–	+	+	+	+
	**Family Megophryidae**											
18	*Leptobrachiumhendricksoni*	+	–	+	+	+	+	+	+	+	+	+
19	*Leptobrachellasola*	–	–	–	–	–	–	+	+	+	+	–
20	*Pelobatrachusnasutus*	–	–	+	–	+	+	–	+	+	+	+
	**Family Microhylidae**											
21	*Kalophrynuskiewi*	–	–	–	–	–	–	+	+	+	+	+
22	*Kalophrynuspalmatissimus*	–	–	–	–	–	–	–	+	–	–	–
23	*Kaloulalatidisca*	–	–	–	–	–	–	+	+	–	+	+
24	*Kaloulapulchra*	–	–	–	–	+	+	+	+	+	–	+
25	*Microhylabedmorei*	–	–	–	–	–	–	+	+	–	–	–
26	*Microhylabutleri*	–	–	–	+	+	+	+	+	+	+	+
27	Microhylacf.heymonsi	+	+	+	+	+	+	+	+	+	+	+
28	*Microhylasuperciliaris*	–	–	–	–	+	–	–	+	–	+	+
29	*Micrylettadissimulans*	–	–	–	–	–	–	–	+	+	+	+
30	*Phrynellapulchra*	–	–	–	–	–	+	–	–	–	–	–
	**Family Ranidae**											
31	*Amolopsgerutu*	+	–	+	+	+	+	+	+	+	+	+
32	*Chalcoranalabialis*	–	–	+	+	+	+	+	+	+	+	+
33	*Humeranamiopus*	–	–	–	–	+	–	+	+	+	+	+
34	*Hylaranaerythraea*	+	+	+	+	+	+	+	+	+	+	–
35	*Indosylvirananicobariensis*	+	–	–	–	+	–	+	+	+	+	+
36	*Odorranahosii*	+	–	+	+	+	+	+	+	+	+	+
37	*Pulchranaglandulosa*	–	–	–	–	+	+	+	+	+	+	+
38	*Pulchranalaterimaculata*	–	–	–	–	–	–	–	–	–	+	+
39	*Pulchranasundabarat*	+	–	+	–	–	–	+	+	+	+	–
40	*Sylviranamalayana*	+	–	+	–	+	+	+	+	+	–	–
	**Family Rhacophoridae**											
41	*Kurixaluschaseni*	–	–	–	–	–	+	–	+	+	+	+
42	*Nyctixaluspictus*	–	–	–	–	–	+	+	+	+	+	+
43	*Polypedatescolletti*	–	–	–	–	–	–	–	–	–	–	+
44	*Polypedatesdiscantus*	–	–	–	–	–	–	+	+	+	+	+
45	*Polypedatesleucomystax*	+	+	+	+	+	+	+	+	+	+	+
46	*Polypedatesmacrotis*	–	–	–	–	+	+	+	+	+	+	+
47	*Rhacophorusnigropalmatus*	–	–	–	–	+	–	–	–	–	+	–
48	*Rhacophoruspardalis*	–	–	–	–	+	–	–	–	–	–	–
49	*Thelodermahorridum*	–	–	–	–	+	–	–	–	–	–	–
50	*Thelodermalicin*	–	–	–	–	–	–	–	+	–	–	–
51	*Zhangixalusprominanus*	–	–	–	–	+	–	–	+	+	+	–
52	*Zhangixalustunkui*	–	–	–	–	+	–	–	–	–	–	–
	Total number of species	**14**	**8**	**14**	**12**	**28**	**20**	**29**	**43**	**34**	**38**	**34**

**Class Amphibia**

**Order Gymnophiona**

### Family Ichthyophiidae

#### ﻿ Ichthyophis cf. asplenius

Taxon classificationAnimaliaGymnophionaIchthyophiidae

B0019DDC-663F-5BDA-B61A-F666467D9BFC

**Figure 2.**
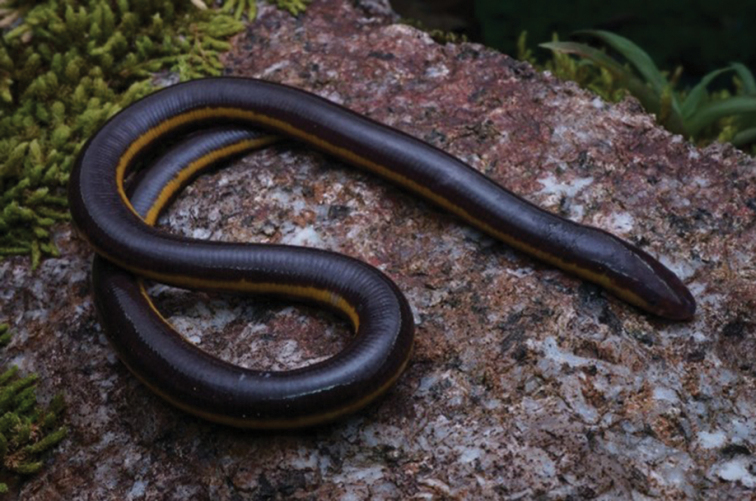
Ichthyophiscf.asplenius.

Fig. 2 Malayan Caecilian

##### Examined specimens.

One specimen was collected from SRF (UMTZC1792, SVL = 198 mm).

##### Identification.

The specimen had elongated and cylindrical body with SVL 198 mm; head as wide as body; snout round; small eyes; body darkish purple with pale yellowish lateral band. Specimen tentatively recognised as Ichthyophiscf.asplenius as suggested by Nishikawa et al. (2012), until further research to reconfirmed taxonomy of this group in Peninsular Malaysia.

##### Remarks.

The specimen was collected from the downstream areas of the Peres River. The collected individual was spotted amongst piles of wet leaves at the stream edge. This species is a new record for the amphibians in Hulu Terengganu.

### Order Anura

#### Family Bufonidae

##### ﻿ Ansonia latiffi

Taxon classificationAnimaliaAnuraBufonidae

Wood, Grismer, Norhayati & Juliana, 2008

4414D779-FF1E-5AA3-A2B0-891CA11D817C

**Figure 3.**
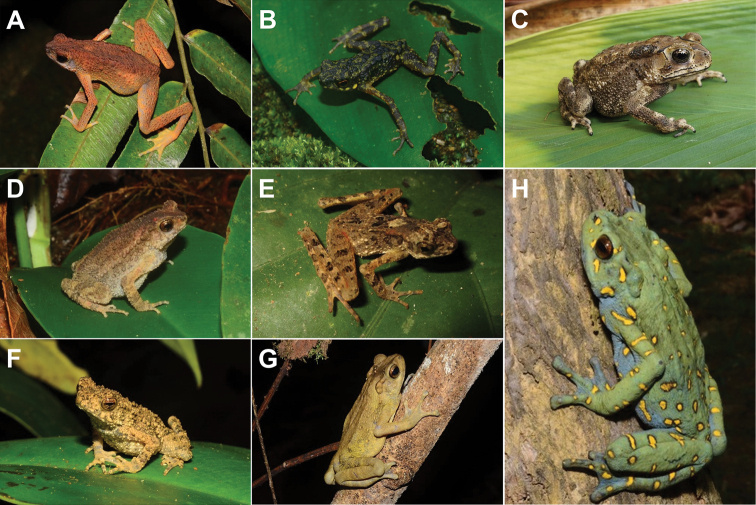
**A***Ansonialatiffi***B***Ansonialumut***C***Duttaphrynusbengalensis* (*Duttaphrynus* sp.1) **D***Ingerophrynusparvus***E***Leptophryneborbonica***F***Phrynoidisasper***G** male *Rentapiaflavomaculata***H** female *R.flavomaculata*.

Fig. 3A Latiff’s Torrent-dwelling Toad

###### Examined specimens.

Eight specimens were collected from SRF consisted of four males (UMTZC1319, UMTZC1400, UMTZC1461, and UMTZC1575, SVL = 38–60 mm) and four females (UMTZC1318, UMTZC1353, UMTZC1401, and UMTZC1553, SVL = 41–59 mm).

###### Identification.

Morphological characters of the specimens from SLF agreed well with the description by Wood et al. (2008b). Size (SVL: 38–60 mm, *n* = 4 males; 41–59 mm, *n* = 4 females); snout projecting beyond lower jaw; tympanum distinct; interorbital ridges absent; small warts at jaw; head narrow in females, but wide in males; inner and outer metatarsal tubercles present; first finger reaching tip of second finger; single mandibular asperities in UMTZC1400 and double for other specimens; dorsal tubercles distinct; spotting at gular region, obscured in UMTZC1353 and UMTZC1553; abdomen finely granular; no dorsolateral row of tubercles;

###### Remarks.

All observed and collected *A.latiffi* were found along the banks of the small streams of the Peres River within the areas of SRF. *Ansonialatiffi* is typically found on rocky substrate or the ground of sloping terrain, and sometimes found perched on low vegetation below 1 m from the ground.

##### ﻿ Ansonia lumut

Taxon classificationAnimaliaAnuraBufonidae

Chan, Wood, Anuar, Muin, Quah, Sumarli & Grismer, 2014

6DDB8FDB-A89E-5B7A-AB81-3513787C98DC

Fig. 3B Mossy Stream Toad

###### Examined specimens.

Three males were collected from SRF (UMTZC1527 and UMTZC1991, SVL = 15–24 mm) and SAP (UMTZC1615, SVL = 26 mm).

###### Identification.

Morphological characters of the specimens agreed well with the description by Chan et al. (2014a). Size (SVL: 15–26 mm, *n* = 3 males); distinct tympanum; snout projecting beyond lower jaw; snout wider than long; paratoid gland absent; interorbital ridges absent; large yellow rectal tubercles behind tympanum; limbs with yellow cross bars; venter surface pale grey with fine white spotting; first finger much shorter than second; no dorsolateral row of tubercles; dorsum blackish with greenish-yellow reticulations; and flank with small yellow spots.

###### Remarks.

*Ansonialumut* was first collected from the Bubu River in SAP by Davis et al. (2016) (LSUHC 11212–13), and a second specimen (UMTZC1615) was collected in the same place during recent surveys. For the Peres River, two specimens were collected from a small stream area (UMTZC1527) and in drift-fenced pitfalls (UMTZC1991). This stream-dwelling species was found on substrates such as granite rock covered with moss, low vegetation at steep edges of the stream, and collected as well in pitfall traps.

##### ﻿ Duttaphrynus bengalensis

Taxon classificationAnimaliaAnuraBufonidae

(Duttaphrynus sp. 1) (Daudin, 1802)

F5EB2B53-3690-51D6-8DB1-CEFBD7B730A0

Fig. 3C Common Asian Toad

###### Examined specimens.

Four males were collected from SRF (UMTZC1147, UMTZC1148, and UMTZC1149, SVL = 47–68 mm) and SAP (UMTZC1065, SVL = 59 mm).

###### Identification.

Morphological characters of the specimens agreed well with the former description by Berry (1975). Size (SVL: 47–68 mm, *n* = 4 males); stout bodies; head with supraorbital and supratympanic bony ridges; parietal ridges absent; snout obtusely pointed; tympanum distinct; tips of digits blunt; subarticular tubercles distinct; toes more than ½ webbed; paratoid gland ellipsoidal; outer metatarsal tubercles smaller than inner metatarsal tubercles. Based the revision by Jablonski et al. (2022), the specimens resembled the characteristics of *Duttaphrynusbengalensis* comb. nov. and “*hazarensis*” such as the concave interorbital space; interorbital space larger than upper eyelid width and internarial distance; snout longer than horizontal eye diameter; tympanum oval; canthus rostralis with a ridge and sharp (Daudin 1802; Khan 2001).

###### Remarks.

This species was more frequently observed at SAP compared to SRF, probably due to the more disturbed and man-made environment that is favoured by this commensal anuran (Shahriza and Ibrahim 2012; Badli-Sham et al. 2019). The species was commonly observed in drains, irrigation ditches, abandoned ponds, and even along the roadside. Calls of this species could be heard after rains. Recently, Jablonski et al. (2022) carried out the molecular assessment on the *Duttaphrynusmelanostictus* complex in the South-East Asia, which revealed the two groups of populations namely, *Duttaphrynusbengalensis* comb. nov. and “*hazarensis*” (*Duttaphrynus* sp. 1) and *Duttaphrynus* sp. 2 sensu Bisht et al. (2021). However, careful inspection of collected specimens shows a resemblance to *Duttaphrynusbengalensis* (*Duttaphrynus* sp. 1) based on five characters by Daudin (1802) and Khan (2001).

##### ﻿ Ingerophrynus parvus

Taxon classificationAnimaliaAnuraBufonidae

(Boulenger, 1887)

FB790FBE-C8D0-51CB-9D20-905DE963F916

Fig. 3D Malayan Dwarf Toad

###### Examined specimens.

Ten specimens were previously collected from SRF consisted of seven males (UMTZC1021, UMTZC1025, UMTZC1026, UMTZC1033, UMTZC1255, UMTZC1339, and UMTZC1398, SVL = 33–37 mm) and three females (UMTZC1023, UMTZC1158, and UMTZC1625, SVL = 42–45 mm).

###### Identification.

Morphological characters of the specimens agreed well with the description by Berry (1975) and Sumarli et al. (2015). Size (SVL: 33–37 mm, *n* = 7 males; 42–45 mm, *n* = 3 females); stout body; head with supraorbital and parietal ridges forming straight lines; supratympanic ridges short; tympanum distinct; snout truncate; finger tips rounded; first finger longer than second; subarticular tubercles distinct; toes ½ webbed; paratoid gland rounded to sub-triangular; dorsum skin with distinct spiny tubercles, venter coarsely granular.

###### Remarks.

Most of the individuals were collected and observed near the stream areas. This species is also common in open recreational areas. *Ingerophrynusparvus* was commonly found on the leaf litter, in rocky crevices and rotten logs, but rarely found on low vegetation.

##### ﻿ Leptophryne borbonica

Taxon classificationAnimaliaAnuraBufonidae

(Tschudi, 1838)

87BCACC0-4298-5D28-8048-D3E7D34CDDB8

Fig. 3E Javan Tree Toad

###### Examined specimens.

Two male specimens were collected from SRF (UMTZC1500 and UMTZC1733, SVL = 22–24 mm).

###### Identification.

Morphological characters of the specimens agreed well with the description by Berry (1975). Size (SVL: 22–24 mm, *n* = 2 males); slender body; head without bony ridges; snout truncate; tympanum distinct; tips of digits swollen into discs; first finger slightly shorter than the second; fingers ½ webbed; subarticular tubercles distinct; skin with small warts; paratoid gland small and almost invisible; dorsum with hourglass shape; pale dorsolateral gland; hind limbs with pinkish red on underside surface.

###### Remarks.

*Leptophryneborbonica* is so far known to occur at the small streams of SRF with the first collected specimen UMTZC1500 from the Peres small stream and UMTZC1733 from the Bubu small stream. This forest-dwelling species was sighted amongst the piles of dead leaves on the forest floor and the banks of the streams.

##### ﻿ Phrynoidis asper

Taxon classificationAnimaliaAnuraBufonidae

(Gravenhorst, 1829)

3223F06F-4AB1-5AFD-8EF3-040CB8D812BA

Fig. 3F Malayan Giant Toad

###### Examined specimens.

Nineteen specimens were collected from SLF consisted of 17 specimens from SRF (Juvenile: UMTZC1136, SVL = 17 mm; Males: UMTZC1020, UMTZC1022, UMTZC1031, UMTZC1083, UMTZC1112, UMTZC1129, UMTZC1135, UMTZC1399, UMTZC1483, UMTZC1508, UMTZC1511, UMTZC1575, UMTZC1576, and UMTZC1577, SVL = 24–111 mm; Females: UMTZC1321 and UMTZC1800, SVL = 93–150 mm) and two specimens from SAP (Males: UMTZC1152 and UMTZC1153, 32–34 mm).

###### Identification.

Morphological characters of the specimens agreed well with the description by Berry (1975) and Sumarli et al. (2015). Size (SVL: 17 mm, *n* = 1 juvenile; 24–111 mm, *n* = 16 males; 93–150 mm, *n* = 2 females); stocky body; supraorbital and supratympanic bony ridges distinct; snout obtusely pointed; paratoid gland rounded to sub-triangular; tympanum distinct; first finger slightly longer than second; subarticular tubercles conspicuous and large. Five specimens absent of X-shaped dorsum marking (UMTZC1031, UMTZC1129, UMTZC1321, UMTZC1483, UMTZC1511).

###### Remarks.

*Phrynoidisasper* is common at the stream areas of SLF. This species was typically observed in rocky crevices and the ground near the stream bank. This toad is also amongst the largest species of anuran recorded in this area with a maximum SVL reaching 150 mm.

##### ﻿ Rentapia flavomaculata

Taxon classificationAnimaliaAnuraBufonidae

Chan, Abraham & Badli-Sham, 2020

177C63F7-5388-5478-AF05-151980723725

Fig. 3G, H Yellow Spotted Tree Toad

###### Examined specimens.

Two adult females were collected from SRF (UMTZC1404 and UMTZC1495, SVL = 102–104 mm).

###### Identification.

Morphological characters of the specimens agreed well with the description of Chan et al. (2020a). Size (SVL: 102–104 mm, *n* = 2 females); moderately stout bodies; head without bony ridges; tympanum distinct; paratoid gland short and distinct; finger tips long and expended into broad discs; inner metatarsal tubercles larger than outer; body greenish with yellow spots and reticulations; venter finely granular. One male from areas near SRF was observed and photographed (Fig. 3G).

###### Remarks.

The first collected specimen UMTZC1404 (Fig. 2H) was from a small stream of the Bubu River, and UMTZC1495 was from a small stream of the Peres River, and the photographed male (Fig. 3G) was from the latter location. Both preserved specimens were collected during the post-monsoon season at SRF. Both were seen on rotten logs and tree trunks within the vicinity of the small streams.

#### Family Dicroglossidae

##### ﻿ Fejervarya limnocharis

Taxon classificationAnimaliaAnuraDicroglossidae

(Gravenhorst, 1829)

29DE2F84-1D5C-524E-ADC8-79ACD3AE2B29

**Figure 4.**
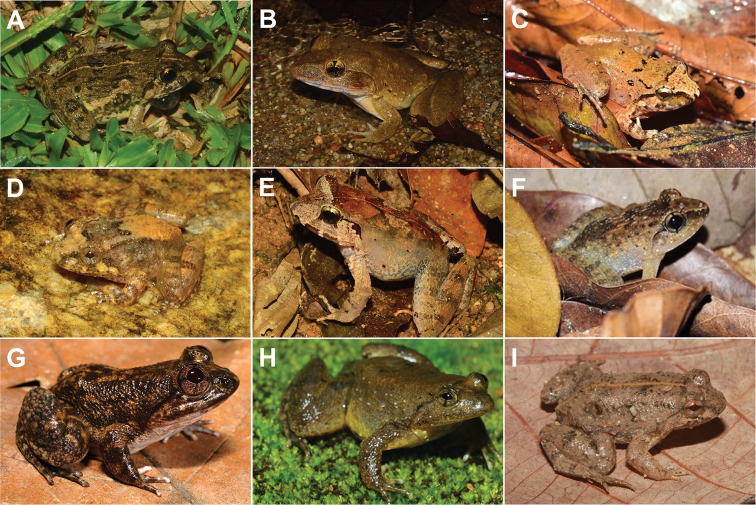
**A***Fejervaryalimnocharis***B***Limnonectesblythii***C***L.hascheanus***D***L.deinodon***E***L.malesianus***F***L.plicatellus***G***L.utara***H***Occidozygasumatrana***I***O.martensii*.

Fig. 4A Rice Field Frog

###### Examined specimens.

Twenty-four specimens were previously collected from SAP (Males: UMTZC1391, UMTZC1396, UMTZC1397, and UMTZC1598, SVL = 14–40 mm; Female: UMTZC1497, SVL = 50 mm), and SRF (Males: UMTZC1002, UMTZC1019, UMTZC1048, UMTZC1049, UMTZC1088, UMTZC1089, UMTZC1150, UMTZC1215, UMTZC1216, UMTZC1229, UMTZC1230, UMTZC1239, and UMTZC1259, SVL = 23–45 mm; Females: UMTZC1003, UMTZC1058, UMTZC1087, UMTZC1122, UMTZC1151, and UMTZC1324, SVL = 49–58 mm).

###### Identification.

Morphological characters of the specimens agreed well with the description of Berry (1975) and Sumarli et al. (2015). Size (SVL: 23–45 mm, *n* = 17 males; 49–58 mm, *n* = 7 females); vomerine teeth in two oblique series between choanae; head moderate; pointed snout; tympanum distinct; supratympanic fold distinct; first finger longer than second; fingers lacking fringes of skin; finger tips blunt; pointed toe tips; inner and outer metatarsal tubercle with oval-shaped; male specimens with nuptial pads on dorsal portion of first finger; dorsum skin with longitudinal skin folds.

###### Remarks.

This species was ubiquitous in cleared and disturbed areas of SLF and is considered as commensal species of frog in this area. Most of the collected specimens were found on the grassy fields and in puddles. Active calling can be heard after the rains.

##### ﻿ Limnonectes blythii

Taxon classificationAnimaliaAnuraDicroglossidae

(Boulenger, 1920)

0C830856-4589-590F-B821-62273629CBB0

Fig. 4B Blyth’s Giant Frog

###### Examined specimens.

Seven specimens were collected from SRF consisted of juveniles (UMTZC1390 and UMTZC1599, SVL = 31–38 mm), males (UMTZC1004 and UMTZC1394, SVL = 84 to 87 mm), and females (UMTZC1393, UMTZC1459, and UMTZC1491, SVL = 48–68 mm).

###### Identification.

Morphological characters of the specimens agreed well with the description by Berry (1975) and Sumarli et al. (2015). Size (SVL: 31–38 mm, *n* = 2 juveniles; 84–87 mm, *n* = 2 males; 48–68 mm, *n* = 3 females); stout body; head long and narrow; pointed snout projecting beyond lower jaw; supratympanic fold distinct; supratympanic fold distinct; toes fully webbed; upper eyelids with low and rounded tubercles; dorsum smooth and sometimes scattered with low tubercles; dorsum pattern variable from dark W-shaped marking on back (UMTZC1390, UMTZC1393, UMTZC1459, UMTZC1491, and UMTZC1599) to a broad vertebral stripe from snout to vent (UMTZC1394), or plain dorsum (UMTZC1004).

###### Remarks.

All specimens were collected from the recreational zones of SLF and the small streams of the Peres and Bubu Rivers. This species is usually found on the ground at stream banks.

##### ﻿ Limnonectes hascheanus

Taxon classificationAnimaliaAnuraDicroglossidae

(Stoliczka, 1870)

1CB2A9EA-DF1A-558E-9260-9DA97344DA21

Fig. 4C Hill Forest Frog

###### Examined specimens.

Two male specimens were collected from SAP (UMTZC1516, SVL = 29 mm) and SRF (UMTZC1529, SVL = 19 mm).

###### Identification.

Morphological characters of the specimens agreed well with the description by Berry (1975) and Hong et al. (2021). Size (SVL: 19–29 mm, *n* = 2 males); vomerine teeth in two oblique oval groups; head moderate; dark crossbar between eyes; rounded snout; tympanum distinct; distinct supratympanic fold from eye to shoulder; inner metatarsal tubercles large; outer metatarsal tubercles absent; digit tips lacking of circum marginal grooves; toes webbed but not reaching discs of second and third toes; dorsum skin smooth with small and low tubercles; dorsolateral fold absent; dorsum colour pale brown with small dark spots and W-shaped marking.

###### Remarks.

UMTZC1516 was collected from hilly terrain at SAP and they are usually seen quietly perched amongst piles of dead leaves. UMTZC1529 was found at the Herbal Park within SRF on similar substrate as the preceding specimen.

##### ﻿ Limnonectes deinodon

Taxon classificationAnimaliaAnuraDicroglossidae

Dehling, 2014

BB86ED60-2915-5145-921E-70A8E6C9AD75

Fig. 4D Flat-headed Corrugated Frog

###### Examined specimens.

Seventeen specimens were collected from SRF consisted of juveniles (UMTZC1386, UMTZC1388, UMTZC1548, and UMTZC1549, SVL = 20–30 mm), males (UMTZC1392, UMTZC1395, UMTZC1458, UMTZC1547, and UMTZC1557, SVL = 43–55 mm) and females (UMTZC1125, UMTZC1370, UMTZC1371, UMTZC1374, UMTZC1387, UMTZC1389, UMTZC1470, and UMTZC1471, SVL = 31 to 53 mm).

###### Identification.

Morphological characters of the specimens agreed well with the description by Dehling (2014). Size (SVL: 20–30 mm, *n* = 4 juveniles; 43–55 mm, *n* = 5 males; 31–53 mm, *n* = 8 females); vomerine teeth in two oblique series behind choanae; head wide and moderately depressed; lower jaw with a pair of prominent odontoids; rounded snout; tympanum hidden; supratympanic fold distinct; digit tips rounded and slightly swollen; first finger shorter than second; fingers with narrow dermal fringes; toes webbing reduced which does not reach toe disc and extend beyond penultimate subarticular tubercle on fourth toe; elongate inner metatarsal tubercle and small outer metatarsal tubercle; dorsum and limbs with longitudinal and corrugated warts; dorsum colour variable from pale brown to bright orange; plain or pale marking on back. This species was previously identified as *L.laticeps* and *L.khasianus* (Ohler and Deuti 2013; Sumarli et al. 2015).

###### Remarks.

The species was ubiquitous at stream areas of SRF and can be found at various microhabitats such as in rocky crevices, dead leaves, rotten logs, or intermittent pools near the streams.

##### ﻿ Limnonectes malesianus

Taxon classificationAnimaliaAnuraDicroglossidae

(Kiew, 1984)

7A81B59C-13B5-5C62-9EAE-DAC7BC3C93DE

Fig. 4E Malayan River Frog

###### Examined specimens.

Two male specimens were collected from SRF (UMTZC1123 and UMTZC1628, SVL= 74–81 mm).

###### Identification.

Morphological characters of the specimens agreed well with the description by Grismer (2012). Size (SVL: 74–81 mm, *n* = 2 males); broad head; rounded snout projecting beyond lower jaw; lower jaws of males with two fang-like projections; upper eyelids with prominent and spiny tubercles; tympanum distinct; supratympanic distinct; dorsum smooth with W-shaped marking, small tubercles, and longitudinal folds. UMTZC1628 had fine and whitish vertebral line from snout to vent and along the upper side of thigh, while UMTZC1123 had no vertebral line on the back.

###### Remarks.

All specimens were collected from a small stream of the Peres River at SRF. Additional individuals of this species can be found on forest floors and stream banks of the small streams and recreational zones in SRF.

##### ﻿ Limnonectes plicatellus

Taxon classificationAnimaliaAnuraDicroglossidae

(Stoliczka, 1873)

E84104C9-F1D4-5E19-8E1F-E2BF55E89387

Fig. 4F Rhinoceros Frog

###### Examined specimens.

Two adult specimens were collected from SRF for male (UMTZC1460, SVL = 35 mm) and female (UMTZC1512, SVL = 35 mm).

###### Identification.

Morphological characters of the specimens agreed well with the description by Berry (1975) and Sumarli et al. (2015). Size (SVL: 35 mm for male and female); large head; blunted snout; vomerine teeth in two oblique series between choanae; lower jaw with two fang-like projections; head with knob-like bony projection between eyes in males; tympanum distinct; supratympanic fold distinct; finger tips dilated into small disc; first finger slightly longer than second; toes 2/3 to 3/4 webbed; subarticular tubercles well developed; inner metatarsal tubercle elongate; outer metatarsal tubercle absent; dorsum skin with longitudinal folds; dorsum colour bronze to reddish brown.

###### Remarks.

UMTZC1460 was collected from a small stream of Peres River, and UMTZC1512 from the recreational zone within SRF. Both were found on the forest litter.

##### ﻿ Limnonectes utara

Taxon classificationAnimaliaAnuraDicroglossidae

Matsui, Belabut & Ahmad, 2014

E979DF79-AC2B-5856-A556-10E0504822F4

Fig. 4G Warty River Frog

###### Examined specimen.

One male specimen was collected from SLF (UMTZC1964, SVL = 49 mm).

###### Identification.

Morphological character of the specimen agreed well with the description by Matsui et al. (2014). Size (SVL: 49 mm, *n* = 1 male); obtusely pointed snout; head longer than broad; tympanum almost visible; supratympanic fold distinct; finger tips bluntly rounded; first finger slightly longer than second; nuptial pad present on first finger and second finger of males; toes webbed at base; subarticular tubercles oval-shaped; inner metatarsal tubercle large; outer metatarsal tubercle absent; dorsum smooth; less densely arranged circum-cloacal warts; tibia surface densely covered by warts; dark blotches absent on rear thigh.

###### Remarks.

Information of *L.utara* from SLF is limited to a specimen and photograph of a dead specimen, as it was contributed through recent undergraduate sampling. This species was previously reported as Limnonectescf.kuhlii in SRF.

##### ﻿ Occidozyga sumatrana

Taxon classificationAnimaliaAnuraDicroglossidae

(Peters, 1877)

6613AE0C-97B3-5252-AF50-8262D03995AA

Fig. 4H Yellow Bellied Puddle Frog

###### Examined specimens.

Seven adult male specimens were collected from SRF (UMTZC1507, UMTZC1561, UMTZC1562, UMTZC1610, UMTZC1629, UMTZC1631, and UMTZC1734SVL, SVL = 22–35 mm).

###### Identification.

Morphological characters of the specimens agreed well with the description by Davis et al. (2018) and Hong et al. (2021). Size (SVL: 22–35 mm, *n* = 7 males); depressed head; rounded snout; tympanum present but not visible through skin; weak supratympanic fold; tips of digits blunt and dilated to small disc; elliptical and compressed inner metatarsal tubercle, outer metatarsal tubercle absent; toes webbed and reaching discs of all toes; dorsum patterns variable from having a broad and pale vertebral stripe between eyes and shoulder (UMTZC1629) to indistinct dark marking (UMTZC1507, UMTZC1561, UMTZC1610, UMTZC1631, and UMTZC1734), dense and dark blotching, or whitish marking on snout and interorbital region (UMTZC1562).

###### Remarks.

All specimens were found in temporary stagnant water bodies near the Peres River at SRF, such as puddles or intermittent pools that developed after heavy rains on the forest floor and near stream areas. The species is typically observed with its body partially submerged in water with eyes exposed.

##### ﻿ Occidozyga martensii

Taxon classificationAnimaliaAnuraDicroglossidae

(Peters, 1867)

D03ABB9A-618A-52E5-93F9-6F868166EE79

Fig. 4I Marten’s Puddle Frog

###### Examined specimens.

No specimen was collected for this species, but it was recorded by field observation and photographs of two individuals at an artificial pond at Herbal Park in SRF (UMTZCP040519–122).

###### Identification.

All observed individuals had stocky body; flattened heads; tympanum covered by skin; supratympanic fold distinct; first finger longer than second; inner and outer metacarpal tubercles distinct; toes completely webbed; dorsum paler brown with numerous dark blotches and indistinct blackish dorsolateral stripe; venter smooth and yellowish white. Morphological features of these individuals closely resemble *Occidozygamartensii* based on photographic material illustrated in Chan et al. (2010) from Pulau Pangkor, Shahriza and Ibrahim (2014) from Ulu Paip Recreational Forest, and in Hong et al. (2021) from Batu Hampar Recreational Forest.

###### Remarks.

Both individuals were observed perched on a leaf overhanging the pond, and they quickly escaped into the water when approached.

#### Family Microhylidae

##### ﻿ Kalophrynus kiewi

Taxon classificationAnimaliaAnuraMicrohylidae

Matsui, Eto, Belabut & Nishikawa, 2017

1846143E-D71D-5559-BFE4-241DA9690985

**Figure 5.**
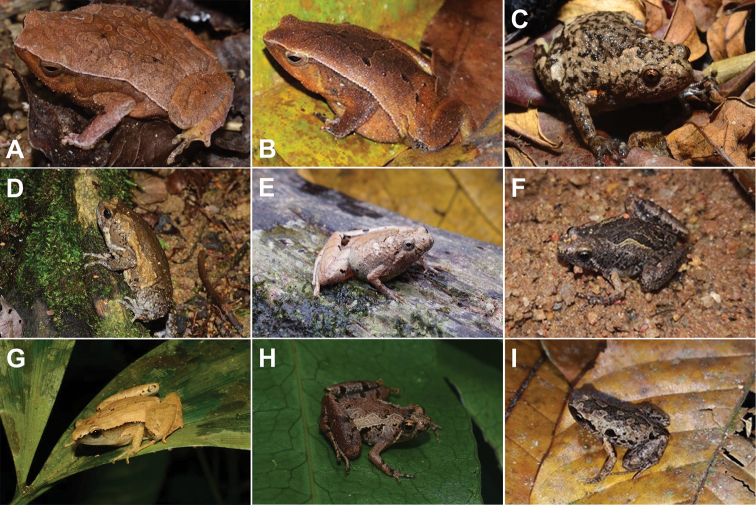
**A***Kalophrynuskiewi***B***K.palmatissimus***C***Kaloulalatidisca***D***K.pulchra***E***Microhylaberdmorei***F***M.butleri***G**M.cf.heymonsi**H***M.superciliaris***I***Micrylettadissimulans*.

Fig. 5A Kiew’s Sticky Frog

###### Examined specimens.

Three specimens were collected from SRF consisted of males (UMTZC1484 and UMTZC1563, SVL 29–36 mm) and female (UMTZC1614, SVL = 43 mm).

###### Identification.

All specimens were previously identified as Kalophrynuscf.pleurostigma and were re-examined following the description of populations from Peninsular Malaysia by Matsui et al. (2017) as *K.kiewi*. Morphological characters of the specimens agreed well with the description of *K.kiewi* in having size (SVL: 29–36 mm, *n* = 2 males; 43 mm, *n* = 1 female); pointed snout which directed downwards; tympanum distinct; first and fourth fingers shorter than second; finger tips rounded and not dilated; fingers with distinct subarticular tubercles (two on third finger and three on other fingers); toes moderately webbed; toes with distinct subarticular tubercles (one on first, second and fifth toes, two on third toe, and three on fourth toe); dorsum skin glandular and spineless; distinct dorsum gland around arm insertion; dorsum pattern with irregular markings extending between eyes and supra-scapular area.

###### Remarks.

All specimens were collected from the trekking trails and artificial pond within the Herbal Park at SRF. No calling was heard, but *K.kiewi* was frequently found on the forest litter and generally towards the monsoon season. This species is a new record for the amphibians in Hulu Terengganu.

##### ﻿ Kalophrynus palmatissimus

Taxon classificationAnimaliaAnuraMicrohylidae

Kiew, 1984

DE4AFC9E-7702-5F62-9EA1-4F234B31FDB3

Fig. 5B Web-footed Sticky Frog

###### Examined specimens.

Two male specimens were collected from SRF (UMTZC1486 and UMTZC1632 = 35–38 mm).

###### Identification.

Morphological characters of the specimens agreed well with the description by Zug (2015). Size (SVL: 35 38 mm, *n* = 2 males); head slightly broader than long; snout moderately broad; tympanum distinct; finger tips rounded and not dilated; fingers with distinct subarticular tubercles; no subarticular tubercles on fifth toe; toes strongly webbed; inner metatarsal tubercle oval-shaped; small and indistinct outer metatarsal tubercle; dorsum pale brown to reddish brown; dorsum patterns with darker hour-glass shaped marking on back.

###### Remarks.

Specimens of this species were collected from the open areas of the camping site, and trekking trail of the Herbal Park at SRF, among leaf litter and rotten logs. Brief calling was heard from a specimen found at the camping site in mid-November. This species is a new record for the amphibians in Hulu Terengganu.

##### ﻿ Kaloula latidisca

Taxon classificationAnimaliaAnuraMicrohylidae

Chan, Grismer & Brown, 2014

2A52CE11-F9C1-5E30-B3CD-5CCA391BEA37

Fig. 5C Wide-Disked Bull Frog

###### Examined specimens.

Five specimens (formerly identified as *K.baleata*) were collected from SRF consisted of juvenile (UMTZC1011, SVL = 20 mm) and adult males (UMTZC1310, UMTZC1316, UMTZC1464, and UMTZC1482, SVL = 35–64 mm).

###### Identification.

Morphological characters of the specimens agreed well with the description of northern populations from Peninsular Malaysia by Chan et al. (2014b). Size (SVL: 20 mm, *n* = 1 juvenile; 35–64 mm, *n* = 4 males); head wider than long; snout obtusely pointed; tympanum hidden; supratympanic fold present; limbs long and robust; digit tips expended into distinct discs lacking of circum-marginal grooves; toes webbed at base; subarticular tubercles distinct; inner metatarsal tubercle large and oval-shaped; outer metatarsal tubercle small and rounded; arm and limb insertion with yellowish patches (pale patches in preserved specimens); dorsum with black speckled marking.

###### Remarks.

*Kaloulalatidisca* was frequently spotted on tree trunks around the open and recreational areas, and occasionally at the small streams of the Peres River at SRF. No calling was heard, but *K.latidisca* was typically found in mid-November and possibly until the end of the monsoon season. This species is a new record for the amphibians in Hulu Terengganu.

##### ﻿ Kaloula pulchra

Taxon classificationAnimaliaAnuraMicrohylidae

Gray, 1831

B0DA81BF-0710-55E3-B6DE-0A658D067692

Fig. 5D Painted Bull Frog

###### Examined specimens.

Four specimens were collected from SRF (Juvenile: UMTZC1414, SVL = 15 mm; Males: UMTZC1082 and UMTZC1415, SVL = 57–62 mm) and SAP (Male: UMTZC1056 = 45 mm).

###### Identification.

Morphological characters of the specimens agreed well with the description by Berry (1975) and Davis et al. (2018). Size (SVL: 15 mm, *n* = juvenile; 45–62 mm, *n* = 3 males); stocky body; snout rounded; tympanum distinct; finger tips with expended discs; toes webbed at base; dorsum skin smooth; dorsum colouration medium to dark brown with broad orange, black-edged, or pale stripes which extend from head along each side of body.

###### Remarks.

*Kaloulapulchra* was ubiquitous in open and recreational areas in SLF, with a tendency to be encountered hiding in irrigation ditches, drains, and toilets. The calls of adult males were heard during and after the heavy rains.

##### ﻿ Microhyla berdmorei

Taxon classificationAnimaliaAnuraMicrohylidae

(Blyth, 1856)

8F2F0D4D-E62D-5B50-91D8-07B66C19944C

Fig. 5E Berdmore’s Narrow-mouthed Frog

###### Examined specimens.

Three male specimens were collected from SRF (UMTZC1457, SVL = 12 mm) and SAP (UMTZC1062 and UMTZC1063, SVL 13–14 mm).

###### Identification.

Morphological characters of the specimens agreed well with the description by Berry (1975), Sumarli et al. (2015) and Garg et al. (2019). Size (SVL: 12–14 mm, *n* =3 males); head as long as broad; snout obtusely pointed; tympanum hidden by skin; finger tips swollen into small discs; toes almost completely webbed; dorsum with a pale stripe from eyes to shoulder; dorsum pattern with broad black marking; anal region with black spots.

###### Remarks.

UMTZC1457 was collected from the recreational zone within SRF in leaf litter, UMTZC1062 and UMTZC1063 were found at the roadside within SAP, also in leaf litter. Additional individuals were observed inside the artificial ponds at the Herbal Park within SRF during the mid-monsoon season.

##### ﻿ Microhyla butleri

Taxon classificationAnimaliaAnuraMicrohylidae

Boulenger, 1900

C6E1F529-E570-5223-87E7-4859271F4612

Fig. 5F Butler’s Narrow-mouthed Frog

###### Examined specimens.

Eight male specimens were collected from SAP (UMTZC1064, UMTZC1217, UMTZC1218, UMTZC1219, UMTZC1220, UMTZC1221, UMTZC1222, and UMTZC1223, SVL = 21–25 mm).

###### Identification.

Morphological characters of the specimens agreed well with the description by Berry (1975) and Garg et al. (2019). Size (SVL: 21–25 mm, *n* = 8 males); rounded snout; tympanum hidden; upper eyelids without dermal tuberculation; tips of digits dilated into small discs bearing circum-marginal grooves; toes webbed at base; subarticular tubercles small; metatarsal tubercles present; head with whitish streak from the eyes to shoulder; dorsum with wavy markings extending from the eyes to posterior region, forming cross bars on hind limbs with pale edges.

###### Remarks.

*Microhylabutleri* was usually found beneath piles of leaf litter and occurs throughout SLF. Despite the fact that all collected specimens were from SAP, many individuals of this species were observed on leaf litter, mostly along roadsides and trekking trails within SRF.

##### ﻿ Microhyla cf. heymonsi

Taxon classificationAnimaliaAnuraMicrohylidae

Vogt, 1911

7FA256CC-D41E-5ED1-9CE3-F9591AC817EF

Fig. 5G Heymon’s Narrow-mouthed Frog

###### Examined specimens.

Nine specimens were collected from SRF (Males: UMTZC1028, UMTZC1067, UMTZC1224, and UMTZC1226, SVL = 17–24 mm; Females: UMTZC1008 and UMTZC1066, SVL = 25–27 mm) and SAP (Males: UMTZC1320 and UMTZC1489, SVL = 17–20 mm; Female: UMTZC1343, SVL = 29 mm).

###### Identification.

Morphological characters of the specimens agreed well with the description by Berry (1975), Garg et al. (2019) and Sumarli et al. (2015). Size (SVL: 17–24 mm, *n* = 6 males; 25–29 mm, *n* = 3 females); rounded snouts, projecting beyond lower jaw; tympanum barely visible; supratympanic fold distinct; tips of digits dilated to form large disc bearing circum-marginal grooves; toes basally webbed; dorsum with pale coloured vertebral stripe, with black marks on each side, and dark stripe on lateral sides from tip of snout until groin; ventral surface of foot is dark brown.

###### Remarks.

Microhylacf.heymonsi was commonly found beneath piles of leaf litter and in rock crevices throughout SLF. This species was also found to occur in similar man-made ponds as with other species of *Microhyla*. Active and loud calling could be heard from this species after rains. The species is considered a commensal species that is tolerant of habitat alteration (Badli-Sham et al. 2019).

##### ﻿ Microhyla superciliaris

Taxon classificationAnimaliaAnuraMicrohylidae

Parker, 1928

CF62D6B6-87D4-501A-BB6D-7CAB67F80788

Fig. 5H Batu Cave’s Narrowed-mouthed Frog

###### Examined specimens.

One specimen was collected from SRF (UMTZC1761, SVL = 18 mm).

###### Identification.

Morphological characters of the specimen agreed well with the description by Berry (1975), Sumarli et al. (2015), and Manthey et al. (2016). Size (SVL = 18 mm, *n* = 1 male); rounded snout; upper eyelid with dermal tubercles; tympanum hidden; first finger much shorter than second; finger tips lacking discs; toe tips dilated into well-developed discs and bearing circum-marginal grooves; toes fully webbed and reaching to discs of all except fourth toe; subarticular tubercles obscured; distinct metatarsal tubercles; dorsum skin smooth with few tubercles.

###### Remarks.

*Microhylasuperciliaris* can be easily mistaken for *M.butleri* that occurs syntopically in the leaf litter at SLF. This species is a new record for the amphibians in Hulu Terengganu.

##### ﻿ Micryletta dissimulans

Taxon classificationAnimaliaAnuraMicrohylidae

Suwannapoom, Nguyen, Pawangkhanant, Gorin, Chomdej, Che & Poyarkov, 2020

3C0A169D-8217-5C62-A8B9-9B594C9D54E1

Fig. 5I Camouflaged Paddy Frog

###### Examined specimens.

Five specimens were collected from SRF consisted of males (UMTZC1519, UMTZC1521 and UMTZC1573, SVL = 18–23 mm) and females (UMTZC1520 and UMTZC1523, SVL = 23–26 mm).

###### Identification.

Morphological characters of the specimens closely resembled the newly described *Micrylettadissimulans* from Saba Yoi District, Songkhla Province, Southern Thailand (Suwannapoom et al. 2020). Size (SVL: 18–23 mm, *n* = 3 males; 23–26 mm, *n* = 2 females); head longer than wide; snout round; interorbital distance two times wider than upper eyelid width; upper lips lacking white patches; tympanum small and barely visible; finger tips rounded; toe tips rounded and weakly dilated into small discs; fingers and toes without webbings; dorsum colour pale to reddish brown; dorsal pattern with merging brown blotches with beige edge; body flanks brown with black spots and whitish mottling.

###### Remarks.

*Micrylettadissimulans* was commonly found at open areas with grass or places with piles of dead leaves. This species is typically found in early November of each year until the end of the monsoon season. This species is a new record for the amphibians in Hulu Terengganu.

##### ﻿ Phrynella pulchra

Taxon classificationAnimaliaAnuraMicrohylidae

Boulenger, 1887

7DA5BB84-1594-59CC-BE27-8EB9F0DBB370

**Figure 6.**
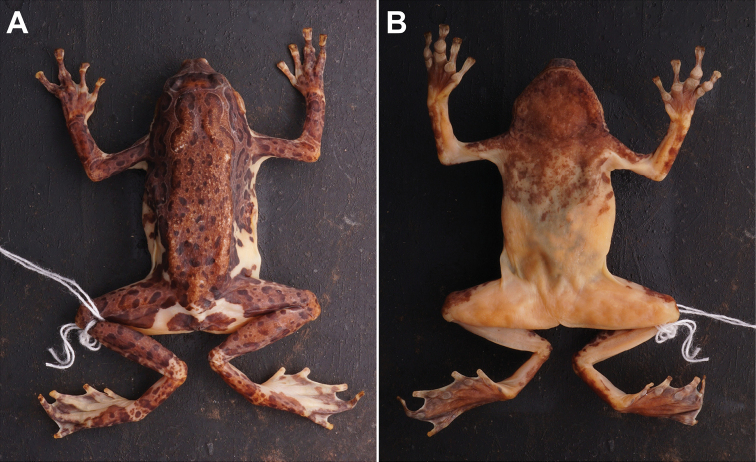
**A** dorsal view and **B** ventral view of *Phrynellapulchra* preserved specimen.

Fig. 6 Malacca’s Narrow-mouthed Frog

###### Examined specimens.

The only female specimen ever collected (UMTZC1302, SVL = 38 mm) which deposited as voucher without much associated information of habitat or site, but with a date of 2014 from SLF.

###### Identification.

Morphological characters of the specimens agreed well with the description by Berry (1975). Size (SVL: 38 mm, *n* = 1 female); small head; snout truncate; tympanum hidden; finger tips depressed and expended into large sub-triangular discs; fingers with distinct subarticular tubercles; toes completely webbed; inner metatarsal tubercles oval-shaped; anal region with large dark spots on that are distinctly separated by white colouration.

###### Remarks.

The available information of this species is limited to a description and photograph of a preserved specimen; however, it is presumed to be found within SRF.

#### Families Megophryidae

##### ﻿ Leptobrachium hendricksoni

Taxon classificationAnimaliaAnuraMegophryidae

Taylor, 1962

ECD3B5DE-036C-54D2-92FB-AE50690AB52F

**Figure 7.**
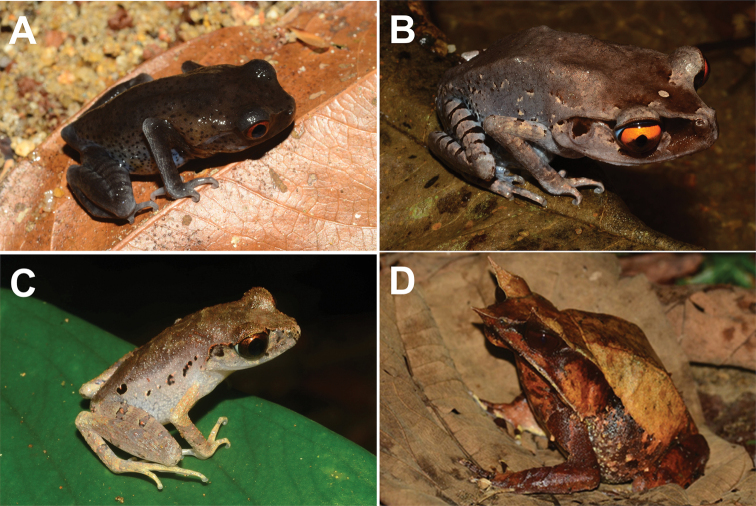
**A** juvenile *Leptobrachiumhendricksoni***B** adult *L.hendricksoni***C***Leptobrachellasola***D***Pelobatrachusnasutus*.

Fig. 7A, B Spotted Litter Frog

###### Examined specimens.

Fourteen specimens were collected from SRF (Juvenile: UMTZC1406, SVL = 28 mm; Males: UMTZC1051, UMTZC1052, UMTZC1091, UMTZC1159, and UMTZC1256, SVL = 40–54 mm; Females: UMTZC1127, UMTZC1160, UMTZC1161, UMTZC1166, and UMTZC1192, SVL = 60–70 mm) and SAP (Juvenile: UMTZC1468, SVL = 35 mm; Males: UMTZC1455 and UMTZC1603, SVL = 40–48 mm).

###### Identification.

Morphological characters of the specimens agreed well with the description by Berry (1975) and Sumarli et al. (2015). Size (SVL: 28–35 mm, *n* = 2 juveniles; 40–54 mm, *n* = 7 males; 60–70 mm, *n* = 5 females); broad head; vomerine teeth absent; tongue notched posteriorly; snout rounded; tympanum distinct; distinct supratympanic fold from eyes to shoulder; tips of digits rounded; first and second finger almost equal in length; two distinct and large metacarpal tubercles; toes ½ webbed; inner metatarsal tubercle oval shaped, outer metatarsal tubercle absent; tibiotarsal joint reaches to shoulder or tympanum; dorsum and venter smooth; dorsum colour dark brown to greyish; venter whitish with black speckling in adults (Fig. 7B). Juvenile specimens (UMTZC1406 and UMTZC1468) had blackish bodies; stumpy tails; and whitish venters with black dots (Fig. 7A).

###### Remarks.

*Leptobrachiumhendricksoni* was common along small streams, cleared areas and man-made ponds in SLF. This species is usually found hiding among grass, on the ground or leaf litter. The tadpoles of *L.hendricksoni* can be found in the small streams, throughout the year.

##### ﻿ Leptobrachella sola

Taxon classificationAnimaliaAnuraMegophryidae

(Matsui, 2006)

791A21C0-6CE3-59CA-B6F3-CBD16DAADDFE

Fig. 7C Spotted Litter Frog

###### Examined specimens.

Eight male specimens were collected from SLF (UMTZC1378, UMTZC1379, UMTZC1408, UMTZC1409, UMTZC1410, UMTZC1411, UMTZC1473, and UMTZC1521, SVL = 20 to 27 mm).

###### Identification.

Morphological characters of the specimens agreed well with the description of *L.sola* by Matsui (2006). Size (SVL: 20–27 mm, *n* = males); head longer than broad; vomerine teeth absent; snout rounded; tympanum distinct; supratympanic fold distinct; finger tips slightly swollen; indistinct subarticular tubercles on fingers; fingers unwebbed with first and fourth almost equal or longer than second; toes basally webbed; tibiotarsal articulation reaching nostril; inner metatarsal tubercle low and small; outer metatarsal tubercle absent; nuptial pads absent; dorsum with indistinct brown markings and blackish blotches on flanks.

###### Remarks.

*Leptobrachellasola* is commonly found near the stream banks, and is usually sighted sitting on low vegetation, bare ground, or piles of dead leaves. This species can be hard to spot during the night as they usually hide amongst the leaf litter and quickly hides beneath the litter when approached.

##### ﻿ Pelobatrachus nasutus

Taxon classificationAnimaliaAnuraMegophryidae

(Schlegel, 1858)

F99DA342-78DC-520C-B213-77196276C7C6

Fig. 7D Malayan Horned Frog

###### Examined specimens.

Three specimens were collected from SLF (Juveniles: UMTZC1103 and UMTZC1187, SVL = 36–38 mm; Male: UMTZC1494, SVL = 94 mm).

###### Identification.

Morphological characters of the specimens agreed well with the description by Berry (1975) and Sumarli et al. (2015). Size (SVL: 36–38 mm, *n* = 2 juveniles; 94 mm, *n* = 1 male); large heads; tongue completely or partly notched posteriorly; vomerine teeth present; snout truncate and projecting beyond the lower jaw; upper eyelids and snout form pointed dermal projection; tympanum distinct; distinct supratympanic fold from eyes to shoulders; subarticular tubercles indistinct; metacarpal tubercles distinct; dorsum smooth with few tubercles; two pairs of longitudinal skin fold on back reaching until the vent; venter smooth with small tubercles.

###### Remarks.

*Pelobatrachusnasutus* were found on the forest floor at various locations: UMTZC1494, UMTZC1103 and UMTZC1187 were collected from the small stream of Peres Rivers where it was sighted on the forest floor. Afterwards, two individuals were observed on the forest floor beside the recreational trail at SRF, and three individuals were observed amongst the dead leaves at the banks of the small stream of the Peres River. Loud callings can be heard typically near the monsoon season.

#### Families Ranidae

##### ﻿ Amolops gerutu

Taxon classificationAnimaliaAnuraRanidae

Chan, Abraham, Grismer & Grismer, 2018

7984B5B8-2125-54CF-B9B8-647B559523DF

**Figure 8.**
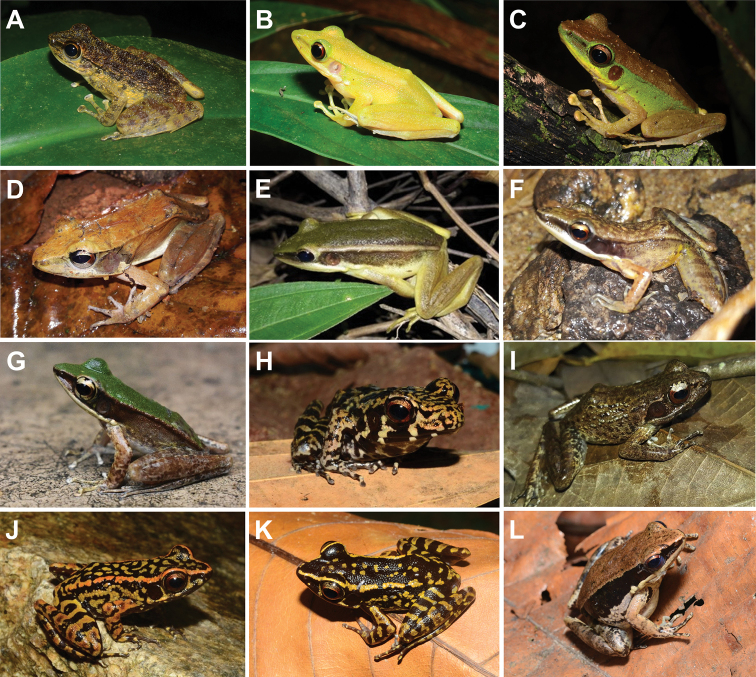
**A***Amolopsgerutu***B***Chalcoranalabialis* (pale yellow colouration) **C***C.labialis* (brown colouration) **D***Humeranamiopus***E***Hylaranaerythraea***F***Indosylvirananicobariensis***G***Odorranahosii***H***Pulchranaglandulosa***I***P.laterimaculata***J***P.sundabarat* (orange dorsolateral stripe) **K***P.sundabarat* (yellow dorsolateral stripe) and **L***Sylviranamalayana*.

Fig. 8A Warty Torrent Frog

###### Examined specimens.

Thirteen specimens were collected from the SLF consisted of adult males (UMTZC1030, UMTZC1032, UMTZC1036, UMTZC1041, UMTZC1043, UMTZC1045, UMTZC1046, UMTZC1106, UMTZC1137, UMTZC1378, and UMTZC1505, SVL = 30–38 mm) and females (UMTZC1297 and UMTZC1377, SVL = 48–53 mm).

###### Identification.

Morphological characters of the specimens agreed well with the description by Chan et al. (2018). Size (SVL: 30–38 mm, *n* = 11 males; 48–53 mm, *n* = 2 females); dorsum densely covered with irregular sized tubercles; dorsolateral region with slightly enlarged, elongated and ridge-like tubercles; dorsal surfaces of hind limb covered with denser and more pronounce tubercles; indistinct pectoral gland with pale yellowish patches.

###### Remarks.

This species can only be found along the torrential zones of the Peres and Bubu Rivers in SLF. The species can be observed perched on the surfaces of boulder stacks along the streams, and occasionally on adjacent low vegetation. Tadpoles of *A.gerutu* can found clinging to boulders below the waterline.

##### ﻿ Chalcorana labialis

Taxon classificationAnimaliaAnuraRanidae

(Boulenger, 1887)

7A265C37-6B9A-56CB-B547-8A8E32F64B35

Fig. 8B, C White-lipped Frog

###### Examined specimens.

Twelve specimens were collected from SRF consisted of one juvenile (UMTZC1032, SVL = 20 mm), males (UMTZC1029, UMTZC1090, UMTZC1235, UMTZC1236, UMTZC1237, UMTZC1383, and UMTZC1384, SVL = 29–36 mm) and females (UMTZC1132, UMTZC1297, UMTZC1317, and UMTZC1622, SVL = 49–55 mm).

###### Identification.

Morphological characters of the specimens agreed well with the description by Berry (1975) and Hong et al. (2021). Size (SVL: 20 mm, *n* = 1 juvenile; 29–36 mm, *n* = 7 males; 49–55 mm, *n* = 4 females); vomerine teeth in oblique groups between choanae; snout pointed; tympanum distinct; digit tips of dilated into discs with circum-marginal grooves; first finger much shorter than second; nuptial pads present on first finger of males; toes webbed and reaching outer edge of first to third toes, inner edge of fifth toe, and fourth toe with one or two phalanges free of webbing; dorsum skin coarsely granular with weak dorsolateral fold; dorsum colour variable from pale green, brownish or pale yellow.

###### Remarks.

This species can be found at many swampy locations and flowing streams within SLF. *Chalcoranalabialis* was usually observed perched on the surface of low vegetation along the streams and swampy areas, and was occasionally found at artificial ponds. All collected and observed individuals were found lower than 2 metres from the ground.

##### ﻿ Humerana miopus

Taxon classificationAnimaliaAnuraRanidae

(Boulenger, 1918)

954F00E6-CEDE-5EAE-8B38-74A04029B538

Fig. 8D Three-striped Frog

###### Examined specimens.

Four specimens were collected from SRF consisted of juvenile (UMTZC1682, SVL = 23 mm), males (UMTZC1007 and UMTZC1379, SVL = 62–65 mm) and female (UMTZC1472, SVL = 70 mm).

###### Identification.

Morphological characters of the specimens agreed well with the description by Berry (1975). Size (SVL: 23 mm, *n* = 1 juvenile; 62–65 mm, *n* = 2 males; 70 mm, *n* = 1 female); vomerine teeth in two oblique series between choanae; snout obtusely pointed; tympanum distinct; finger tips expended into small discs bearing circum-marginal grooves; first finger much longer than second; distinct subarticular tubercles on fingers and toes; toes 2/3 to ¾ webbed with two phalanges of fourth toe free from webbing; inner metatarsal tubercles elliptic; outer metatarsal tubercles indistinct or absent; skin smooth with dorsolateral fold; dorsum with two to three diagonal lines on mid-dorsum region.

###### Remarks.

*Humeranamiopus* was common at the artificial ponds of the Herbal Park in SRF. The species was frequently observed perched on low vegetation beside the artificial ponds, and quickly leapt into the water when disturbed.

##### ﻿ Hylarana erythraea

Taxon classificationAnimaliaAnuraRanidae

(Schlegel, 1837)

58308B12-ACED-567C-91CA-B07F08D5C4E4

Fig. 8E Green Paddy Frog

###### Examined specimens.

Eight specimens were collected from SAP consisted of juvenile (UMTZC1264, SVL = 25 mm), males (UMTZC1182, UMTZC1183, UMTZC1184, and UMTZC1186, SVL = 30–40 mm) and females (UMTZC1050, UMTZC1055, and UMTZC1165, SVL = 42–73 mm).

###### Identification.

Morphological characters of the specimens agreed well with the description by Berry (1975) and Davis et al. (2018). Size (SVL: 25 mm, *n* = 1 juvenile; 30–40 mm, *n* = 4 males; 42–73 mm, *n* = 3 females); vomerine teeth in two oblique group between choanae; head slightly longer than broad; snout pointed; tympanum distinct; weak supratympanic fold; digit tips of expanded into disc bearing circum-marginal grooves; first finger longer than second; toes webbed reaching base of disc in first to third toes, inner edge of fifth, and fourth toes with two phalanges free; inner metatarsal tubercles oval shaped; outer metatarsal tubercles rounded; dorsum smooth with distinct dorsolateral fold; dorsum colour brown with a pale-coloured dorsolateral stripe and brown flanks; ventral surface white.

###### Remarks.

*Hylaranaerythraea* was common in the swampy areas in SAP. The species was also observed by drains, artificial ponds, and sometimes on grassy areas.

##### ﻿ Indosylvirana nicobariensis

Taxon classificationAnimaliaAnuraRanidae

(Stoliczka, 1870)

8D09C583-8993-50B7-8375-165823595CC5

Fig. 8F Nicobar Island Frog

###### Examined specimens.

Three specimens were collected from SRF consisted of one male (UMTZC1571, SVL = 50 mm) and two females (UMTZC1563 and UMTZC1676, SVL = 42–43 mm).

###### Identification.

Morphological characters of the specimens agreed well with the description by Berry (1975) and Shahriza and Ibrahim (2014). Size (SVL: 50 mm, *n* =1 male; 42–43 mm, *n* = 2 females); vomerine teeth in two oblique series between choanae; head longer than wide; snout pointed; tympanum distinct; supratympanic fold absent; first finger longer (UMTZC1571) or equal (UMTZC1563) with second; digit tips expended into small discs bearing circum-marginal grooves; nuptial pad present on first fingers in males; dorsum skin granular; narrowed dorsolateral folds; gravid female with eggs on translucent side of belly.

###### Remarks.

This species was common at the Herbal Park in SRF. The species can be observed perching on rock surfaces at artificial ponds and is abundant at the start of monsoon season. *Indosylvirananicobariensis* is a pond-breeding frog and can inhabit both natural and altered habitats (Lalremsanga et al. 2016).

##### ﻿ Odorrana hosii

Taxon classificationAnimaliaAnuraRanidae

(Boulenger, 1891)

54E96390-82DA-5856-94FF-DC96AF6324AC

Fig. 8G Poisonous Rock Frog

###### Examined specimens.

Twenty adult specimens were collected from SRF (Males: UMTZC1164, UMTZC1170, UMTZC1171, UMTZC1172, UMTZC1173, UMTZC1174, UMTZC1233, UMTZC1299, and UMTZC1304, SVL = 32–66 mm; Females: UMTZC1009, UMTZC1044, and UMTZC1063, SVL = 54–96 mm) and SAP (Males: UMTZC1348, UMTZC1351, UMTZC1385, and UMTZC1504, SVL = 32–50 mm; Females: UMTZC1306, UMTZC1323, UMTZC1380, and UMTZC1481, SVL = 48–98 mm).

###### Identification.

Morphological characters of the specimens agreed well with the description by Berry (1975) and Hong et al. (2021). Size (SVL: 32–66 mm, *n* = 13 males; 48–98 mm, *n* = 7 females); vomerine teeth in two oblique series behind choanae; head as long as broad with pointed snout; tympanum distinct; supratympanic fold; tips of digits expanded into large discs with circum-marginal grooves; first finger equal or shorter than second, and all marked with narrow fringes of skin; nuptial pads on first fingers of males; broad webbing reaching tips of all toes; dorsum skin smooth with weak dorsolateral fold.

###### Remarks.

*Odorranahosii* was ubiquitous at the rocky sections of streams with many boulders. All individuals were collected at night but specimens could be observed in the day hiding in the roots of large trees at the stream bank.

##### ﻿ Pulchrana glandulosa

Taxon classificationAnimaliaAnuraRanidae

(Boulenger, 1882)

EB8CD7EE-3DA3-506D-A7DC-14940B11589E

Fig. 8H Poisonous Gland Frog

###### Examined specimens.

Four male specimens were collected from SRF (UMTZC1301, UMTZC1350, UMTZC1576, and UMTZC1608, SVL = 43–74 mm).

###### Identification.

Morphological characters of the specimens agreed well with the description by Berry (1975) and Sumarli et al. (2015). Size (SVL: 43–74 mm, *n* = 4 males); vomerine teeth in two oblique series between choanae; head large, rounded snout; tympanum distinct; weak supratympanic fold; digit tips dilated into small discs bearing circum-marginal grooves; first finger much longer than second; skin fringes absent on fingers; toes webbing not well-developed; inner metatarsal tubercles oval-shaped; outer metatarsal tubercles small and rounded; dorsum without dorsolateral fold; dorsum surfaces covered with low and rounded glandular warts; dorsum colour greyish to dark brown with indistinct dark blotches; limbs with dark cross bars.

###### Remarks.

*Pulchranaglandulosa* was found in the small streams and recreational areas of SLF. The species was commonly observed hidden amongst piles of dead leaves and rotten logs.

##### ﻿ Pulchrana laterimaculata

Taxon classificationAnimaliaAnuraRanidae

(Barbour & Noble, 1916)

526A82C2-C220-5417-9B15-A71263C2CFF8

Fig. 8I Side-spotted Swamp Frog

###### Examine specimens.

One specimen of adult female specimen was collected from SLF (UMTZC1699, SVL = 50 mm).

###### Identification.

Morphological characters of the specimen agreed well with the description by Sumarli et al. (2015) and Leong et al. (2003). Size (SVL: 50 mm, *n* = 1 female); vomerine teeth in two oblique rows bounded by choanae; head moderate; relatively rounded snout; tympanum distinct and entirely black; upper lips with uninterrupted white line; finger and toe tips expended into discs; toes well-developed webbing but not reaching medial subarticular tubercles; dorsum and flanks with raised rounded tubercles, forming discontinuous longitudinal ridges; distinct humeral glands in male; dorsum pale brown.

###### Remarks.

*Pulchranalaterimaculata* can be found in areas similar to *P.glandulosa* in SLF. The species was commonly observed hidden among piles of dead leaves and rotten logs.

##### ﻿ Pulchrana sundabarat

Taxon classificationAnimaliaAnuraRanidae

Chan, Abraham, Grismer & Brown, 2020

C0DD3050-5D48-5BC0-AADD-416E2D23B8FA

Fig. 8J, K Western Sunda Spotted Stream Frog

###### Examine specimens.

Four specimens were collected from SRF consisted of three males (UMTZC1376, UMTZC1377, and UMTZC1387, SVL = 40 mm) and one female (UMTZC1375, SVL = 57 mm).

###### Identification.

Morphological characters of the specimens agreed well with the description by Chan et al. (2020c). Size (SVL: 40 mm, *n* = 3 males; 57 mm, *n* = 1 female); head longer than wide; snout pointed; tympanum distinct; supratympanic fold absent; digit tips slightly expended into small disc with circum-marginal groove; nuptial pads distinctly separated on first finger in UMTZC1376 and UMTZC1377, and slightly joined in UMTZC1387; toes slightly more than ½ webbed; dorsum smooth and indistinctly glandular; dorsum colour black; dorsum patterns with conspicuous and defined yellowish to bright orange dorsolateral stripe, dorsum and flanks with yellowish blotches; humeral gland raised and blackly pigmented in males; throat and abdomen with white spots.

###### Remarks.

Chan et al. (2020b, c) revised this complex and suggested that the *Pulchranapicturata* from the Malay Peninsula and Sumatra belong to this new species, *P.sundabarat* that is genetically distinct from the true Bornean *P.picturata.* The specimens were all found at the pristine areas in SRF. This species usually hide beneath the roots of large trees on stream banks. The distinct calls of males could be easily heard in those areas.

##### ﻿ Sylvirana malayana

Taxon classificationAnimaliaAnuraRanidae

Sheridan & Stuart, 2018

80BCB3B3-994F-5589-AEDB-4F013CDAB250

Fig. 8L Malayan Black-striped Frog

###### Examined specimens.

Seven specimens were collected from SRF (Males: UMTZC1013, UMTZC1014, UMTZC1107, and UMTZC1382, SVL = 43–47 mm; Females: UMTZC1093 and UMTZC1381, SVL = 51–58 mm) and SAP (Males: UMTZC1308, SVL = 45 mm).

###### Identification.

Morphological characters of the specimens agreed well with the description by Sheridan and Stuart (2018). Size (SVL: 43–47 mm, *n* = 5 males; 51–58 mm, *n* = 2 females); head longer than wide; snout obtusely pointed; tympanum distinct; triangular or teardrop shaped fold slightly behind the tympanum; supratympanic fold absent; digit tips of expended into discs with circum-marginal grooves; first finger much longer than second; toes with well-developed webbing; distinct subarticular tubercles; elongated inner metatarsal tubercle; rounded outer metatarsal tubercle; dorsum finely granular; dorsum brown with broad dark band extending from snout to groin.

###### Remarks.

*Sylviranamalayana* can be found in the disturbed areas of SLF. This species was commonly observed hiding between rock crevices within the Herbal Park.

#### Families Rhacophoridae

##### ﻿ Kurixalus chaseni

Taxon classificationAnimaliaAnuraRhacophoridae

(Smith, 1924)

1C6472F1-67C0-58AF-93E3-B5D08430CE1C

**Figure 9.**
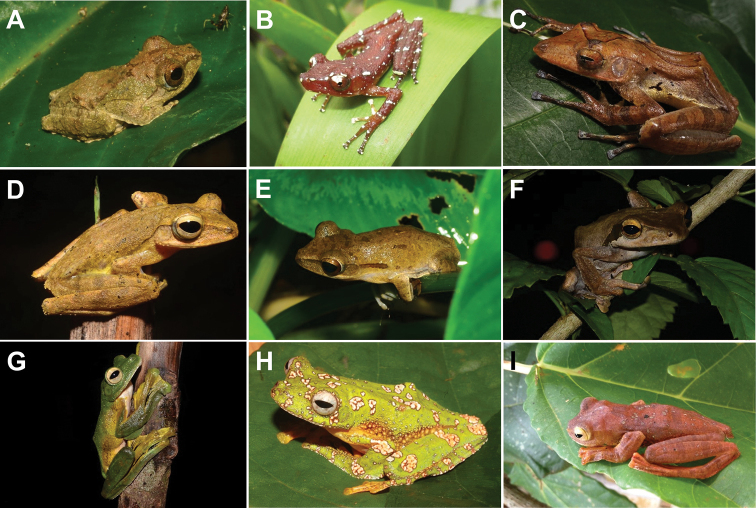
**A***Kurixaluschaseni***B***Nyctixaluspictus***C***Polypedatescolletti***D***P.discantus***E***P.leucomystax***F***P.macrotis***G** adult *Rhacophorusnigropalmatus***H** subadult *R.nigropalmatus***I***R.pardalis*.

Fig. 9A Malay Frilled Tree Frog

###### Examined specimens.

Ten adult specimens were collected from SRF (Males: UMTZC1095, UMTZC1345, UMTZC1360, UMTZC1361, UMTZC1463, UMTZC1465, UMTZC1574, and UMTZC1627, SVL = 30–41 mm; Female: UMTZC1359, SVL = 44 mm) and SAP (Male: UMTZC1502, SVL = 22 mm).

###### Identification.

Morphological characters of the specimens agreed well with the description by Berry (1975) and Sumarli et al. (2015). Size (SVL: 22–41 mm, *n* = 9 males; 44 mm, *n* = 1 female); vomerine teeth at the anterior edges of choanae; head longer than wide; rounded snout with conical projection on tip; tympanum distinct; digit tips dilated into small discs bearing circum-marginal grooves; fingers webbed at base; toes broadly webbed; fourth fingers, tarsus, heel and vent with crenulated dermal fringes; dorsum smooth and slightly granular; dorsum colour pale brown to mossy green.

###### Remarks.

*Kurixaluschaseni* can be found perched on low vegetation within artificial ponds of the Herbal Park. Active calls of males can be heard from this place during the monsoon season in SLF.

##### ﻿ Nyctixalus pictus

Taxon classificationAnimaliaAnuraRhacophoridae

(Peters, 1871)

E4785C4B-4791-591F-A05C-85301843EA3D

Fig. 9B Cinnamon Tree Frog

###### Examined specimens.

Three adult male specimens were collected from SRF (UMTZC1425 and UMTZC1426, SVL = 29–30 mm) and SAP (UMTZC1601, SVL = 35 mm).

###### Identification.

Morphological characters of the specimens agreed well with the description by Berry (1975). Size (SVL: 29–35 mm, *n* = 3 males); no vomerine teeth; head longer than broad; snout obtusely pointed; vertical loreal region; tympanum distinct; digit tips expanded into round or oval-shaped discs; inner metatarsal tubercle oval-shaped, outer metatarsal tubercle absent; dorsum bright orange with whitish spots scattered over the body; limbs with rows of whitish spots forming cross-bars; abdomen with greenish black reticulation.

###### Remarks.

UMTZC1601 was found on low vegetation, less than 1 m from the ground near the suspension bridge at SAP, and UMTZC1426 and UMTZC1425 were found at bushy areas within a small stream off the Peres River in SRF. This species is a new record for the amphibians in Hulu Terengganu.

##### ﻿ Polypedates colletti

Taxon classificationAnimaliaAnuraRhacophoridae

(Boulenger, 1890)

4ACECE5B-5763-5C8F-ABDA-88762BDB540E

Fig. 9C Collett’s Tree Frog

###### Examined specimen.

One adult male specimen was collected from SAP (UMTZC1871, SVL = 67 mm).

###### Identification.

Morphological characters of the specimen agreed well with the description by Berry (1975) and Rujirawan et al. (2013). Size (SVL: 67 mm, *n* = 1 male); triangular head; snout acutely pointed; low tubercles around eyes; tympanum distinct; skin of head not co-ossified with skull; skin coarsely granular; small white spots on rear thigh; heel with a distinct conical tubercle; dorsum pattern with an hour-glass marking extending from interorbital region to back, flanks with black vermiculations.

###### Remarks.

This species was found at the waterfall of SAP, perched on low vegetation less than 2 m from the ground.

##### ﻿ Polypedates discantus

Taxon classificationAnimaliaAnuraRhacophoridae

Rujirawan, Stuart & Aowphol, 2013

B4085EB7-7C62-5634-A098-67D5B80DBB7F

Fig. 9D Malayan Slender Tree Frog

###### Examined specimens.

Twenty-seven specimens were collected from SLF consisted of males (UMTZC1012, UMTZC1015, UMTZC1016, UMTZC1024, UMTZC1081, UMTZC1094, UMTZC1096, UMTZC1113, UMTZC1115, UMTZC1185, UMTZC1240, UMTZC1273, UMTZC1421, UMTZC1509, UMTZC1510, UMTZC1589, and UMTZC1592, SVL = 40–53 mm) and females (UMTZC1006, UMTZC1027, UMTZC1157, UMTZC1179, UMTZC1241, UMTZC1423, UMTZC1424, UMTZC1496, UMTZC1590, and UMTZC1591, SVL = 60–71 mm).

###### Identification.

Based on description by Rujirawan et al. (2013), 29 specimens were identified as *P.discantus* for having size (SVL: 40–53 mm, *n* = 17 males; 60–71 mm, *n* = 10 females); triangular head; snout obtusely pointed; skin of head not co-ossified with skull; tympanum distinct; supratympanic fold distinct; digit tips with well-developed disc bearing circum-marginal groove; fingers webbed at base; toes fully webbed; inner metatarsal tubercle oval-shaped; outer metatarsal tubercle absent; nuptial pad present on first and second fingers in males; rear thigh with indistinct or absence of white spots; heel with rounded tubercle; dorsum with variable patterns: plain dorsum with scattered dark blotches, 2 to 4 longitudinal stripes, or X-shaped marking on interorbital region.

###### Remarks.

This species was common in both natural and man-made habitats of SLF. Most of the collected and observed individuals were found clinging to shrubs or leaves of low vegetation less than 2 m from the ground. This species is a new record for the amphibians in Hulu Terengganu.

##### ﻿ Polypedates leucomystax

Taxon classificationAnimaliaAnuraRhacophoridae

(Gravenhorst, 1829)

6048A0BE-512C-590A-93DF-6A83D11B65E5

Fig. 9E Four-lined Tree Frog

###### Examined specimens.

Twenty-seven specimens were collected from SLF consisted of males (UMTZC1010, UMTZC1114, UMTZC1128, UMTZC1131, UMTZC1180, UMTZC1181, UMTZC1243, UMTZC1244, UMTZC1246, UMTZC1328, UMTZC1329, UMTZC1422, UMTZC1485, UMTZC1624, and UMTZC1417, SVL = 40–52 mm) and females (UMTZC1125, UMTZC1130, UMTZC1154, UMTZC1155, UMTZC1156, UMTZC1178, UMTZC1245, UMTZC1263, UMTZC1272, UMTZC1344, UMTZC1347, and UMTZC1444, SVL = 44–67 mm).

###### Identification.

Morphological characters of the specimens agreed well with the description by Berry (1975) and Sumarli et al. (2015). Size (SVL: 40–52 mm; *n* = 15 males; 44–67 mm, *n* = 12 females); vomerine teeth in between choanae; head longer than broad; rounded snout; tympanum distinct; supratympanic fold present; digit tips expended into large discs with circum-marginal grooves; fingers without webbing; toes fully webbed; forearm with whitish skin flaps; dorsum skin smooth with colour pale to dark tan; dorsum pattern variable with two to four longitudinal stripes or plain with dark blotches.

###### Remarks.

*Polypedatesleucomystax* can be distinguished from *P.discantus* by having the skin of the head fused with the skull, distinct white spots or reticulations on a dark background on the rear thigh, and the absence of a tubercle at the heel. This species known as a commensal species and inhabits all manner of human-made and natural habitats in SLF. This species can be observed clinging onto shrubs or the leaves of low vegetation up to 2.5 m off the ground.

##### ﻿ Polypedates macrotis

Taxon classificationAnimaliaAnuraRhacophoridae

(Boulenger, 1891)

CCF832FD-5E43-5560-A538-3D6B5D55A417

Fig. 9F Dark-eared Tree Frog

###### Examined specimens.

Nine specimens were collected from SRF consisted of males (UMTZC1327, UMTZC1346, UMTZC1418, UMTZC1419, and UMTZC1497, SVL = 60–68 mm) and females (UMTZC1030, UMTZC1097, UMTZC1416, and UMTZC1454, SVL= 62 to 102 mm).

###### Identification.

Morphological characters of the specimens agreed well with the description by Berry (1975) and Sumarli et al. (2015). Size (SVL = 60–68 mm, *n* = 5 males; 62 to 102 mm, *n* = 4 females); vomerine teeth in transverse or slightly oblique series between choanae; head broader than long; rounded snout; tympanum distinct and covered with broad dark stripes; supratympanic fold distinct; digit tips expanded into large discs bearing circum-marginal grooves; fingers free of webbing; nuptial pads present on dorsal portion of first and second fingers; toes fully webbed; dorsum with variable markings from two broad longitudinal stripes (UMTZC1327, UMTZC1419, and UMTZC1497), scattered dark blotches (UMTZC1346, UMTZC1416, and UMTZC1418), plain dorsum (UMTZC1030, UMTZC1097, UMTZC1346, and UMTZC1454), and combination of plain and scattered dark blotches (UMTZC1418).

###### Remarks.

*Polypedatesmacrotis* is restricted to natural or man-made stagnant water bodies closer to the forested areas. This species can also be observed perched on low vegetation near the ponds in syntopy with other species of *Polypedates*.

##### ﻿ Rhacophorus nigropalmatus

Taxon classificationAnimaliaAnuraRhacophoridae

Boulenger, 1895

B5E560DF-2F87-5E8A-92D4-298744870BD4

Fig. 9G, H Wallace’s Flying Frog

###### Examined specimens.

Two specimens were collected from SRF consisted of subadult male (UMTZC1732, SVL = 60 mm) and adult male (UMTZC1057, SVL = 95 mm).

###### Identification.

Morphological characters of the specimens agreed well with the description by Berry (1975). Size (SVL: 60 mm for subadult male; 95 mm for adult male); vomerine teeth in two straight or transversely curved rows between choanae; head longer than broad; rounded snout; tympanum distinct; supratympanic fold absent; digit tips expanded into large and oval-shaped discs and bearing circummarginal grooves; fingers and toes fully webbed; inner metatarsal tubercle oval-shaped; outer metatarsal tubercle absent; dorsum with small clusters of whitish tubercles; broad skin flaps along forearm, rounded on heels, and awning-like flaps on anal region. Head, body, and limbs of subadult male (UMTZC1732) (Fig. 9H) covered with white patches edged in pale brown.

###### Remarks.

UMTZC1057 was collected from a fallen large tree at the Herbal Park within SRF while the second individual UMTZC1732 was found perched on low vegetation at the same location. This species was only encountered during the monsoon season in SLF.

##### ﻿ Rhacophorus pardalis

Taxon classificationAnimaliaAnuraRhacophoridae

Günther, 1858

4B577E8E-3E2A-5F6B-9834-F475EF8DB7E8

Fig. 9I Harlequin Tree Frog

###### Examined specimens.

Two male specimens were collected from SRF (UMTZC1111 and UMTZC1110, SVL = 49–53 mm).

###### Identification.

Morphological characters of the specimens agreed well with the description by Berry (1975) and Harvey et al. (2002). Size (SVL: 49–53 mm, *n* = 2 males); vomerine teeth in two or slightly oblique series at inner edges of choanae; head equally longer with width; snout obtusely pointed; tympanum distinct; supratympanic fold reaching angle of jaws; fingers and toes fully webbed; broad skin flaps along forearm, rounded on heels, and absence on anal region; flanks and abdomen of UMTZC1111 displayed black reticulation, but pale orange in UMTZ1110.

###### Remarks.

Both UMTZC1111 and UMTZC1110 were collected from the large trees beside the artificial pond of the camping site in SRF.

##### ﻿ Theloderma licin

Taxon classificationAnimaliaAnuraRhacophoridae

McLeod & Norhayati, 2007

D4E8A669-FFFC-5E20-849E-AD9E3BD7F5BB

**Figure 10.**
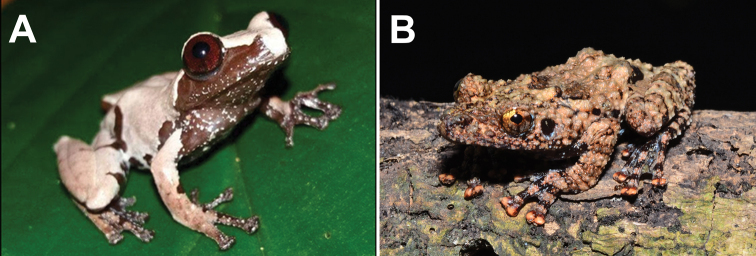
**A***Thelodermalicin* and **B***T.horridum*.

Fig. 10A Smooth-skinned Warted Tree Frog

###### Examined specimens.

One male specimen was collected from SAP (UMTZC1490, SVL = 28 mm).

###### Identification.

Morphological characters of the specimen agreed well with the description by McLeod and Norhayati (2007). Size (SVL: 28 mm, *n* = 1 male); head equally longer with wide; snout obtusely pointed; tympanum distinct; dorsal and lateral surfaces with fine pearly tipped tubercles; coarsely granular venter; no vomerine teeth; tympanum distinct; supratympanic fold reaching angle of jaws; digit tips expended into large disc bearing circum-marginal grooves; fingers webbed at base; toes fully webbed; inner metatarsal tubercle oval-shaped; outer metatarsal tubercle absent; nuptial pad present on second fingers in males; dorsum colour changed from whitish to pale brown when stressed; inguinal area with dark brown blotches.

###### Remarks.

*Thelodermalicin* was found resting on the ground within piles of dead leaves. This species is a new record for the amphibians in Hulu Terengganu.

##### ﻿ Theloderma horridum

Taxon classificationAnimaliaAnuraRhacophoridae

(Boulenger, 1903)

F56BE964-C3CE-53DE-96F0-904433DF9E8B

Fig. 10B Malayan Warted Treefrog

###### Examined specimens.

**Figure 11.**
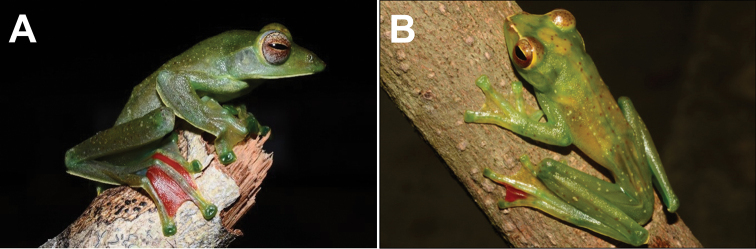
**A***Zhangixalusprominanus* and **B***Z.tunkui*.

No specimen was collected, but photographs of this species from from previous fieldwork in 2014 in SRF (UMTZCP070614-392, Fig. 11B).

###### Identification.

Examined photographs show similar characteristics as described by Berry (1975) and Sumarli et al. (2015) for having stocky body; head wide; distinct tympanum; digit tips expended into distinct disc bearing circum-marginal groove; fingers ½ webbed; toes fully webbed; dorsum rough with warts bearing granular asperities; large lumbar spot; and venter with black-white reticulations.

###### Remarks.

This species was found among the dense vegetation on steep terrain in the forested areas in SRF. *Thelodermahorridum* was spotted clinging to the side of a tree trunk facing upwards.

##### ﻿ Zhangixalus prominanus

Taxon classificationAnimaliaAnuraRhacophoridae

(Smith, 1924)

7B744512-A32D-5A98-93FD-5479C8D973AA

Fig. 11A Green Tree Frog

###### Examined specimens.

Two male specimens were collected from SRF (UMTZC1469 and UMTZC1299, SVL = 56–57 mm).

###### Identification.

Morphological characters of the specimens agreed well with the description by Berry (1975). Size (SVL: 56–57 mm, *n* = 2 males); vomerine teeth in two transverse series between choanae; head broader than long; snout obtusely pointed; tympanum distinct; supratympanic fold hidden; tips of digits expanded into large and oval discs with circum-marginal grooves; fingers and toes fully webbed; broad skin flaps along forearm, rounded on heels, and awning-like skin flaps on anal region.

###### Remarks.

Two specimens of *Zhangixalusprominanus* were collected from the flooded rock pools within the recreational zones of SRF. Another observed individual was also seen perched on low vegetation or rock surfaces in the same area, only found during the monsoon season. This species is a new record for the amphibians in Hulu Terengganu.

##### ﻿ Zhangixalus tunkui

Taxon classificationAnimaliaAnuraRhacophoridae

Kiew, 1987

D6D01E01-6013-55F6-99B6-5AFC2520A2EC

Fig. 11B Johore Flying Frog

###### Examine specimens.

UMTZCP110414-419.

###### Identification.

One individual of *Zhangixalustunkui* was previously recorded in SRF. However, it was only photographed (UMTZCP110414-419, Fig. 11B) and released back to the wild for it was mistaken to be an individual of *Z.prominanus*, which was only later identified as *Z.tunkui* by Evan S.H. Quah. Morphology of photographed individual matched description by Kiew (1987) and Leong (2004) based on toe webbing with reddish colouration between fourth and fifth toes (vs. third to fifth toes in *Z.prominanus*). Photographed individual had body with pale and translucent green colouration; dorsum scattered with whitish spots; smaller SVL, less than 50 mm (M. Taufik Awang, pers. comm.); skin translucent; absence of skin flaps on along forearm; whitish line along the snout, canthus rostralis and around eyes.

###### Remarks.

The individual of this species was found at the flooded rock pools in SRF. This species is a new record for the amphibians in Hulu Terengganu.

**Figure 12.**
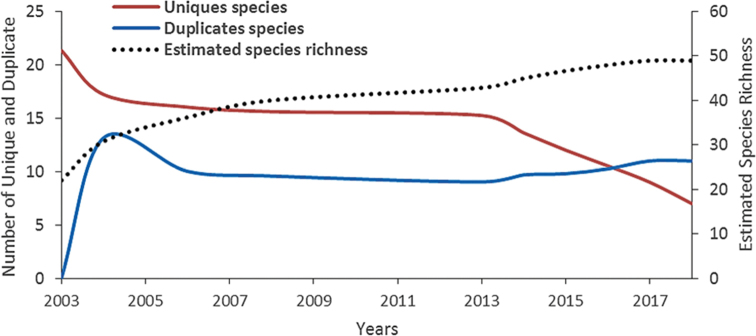
The cumulative species discovery curve of the species (dotted line), species accumulation curve of replicated samples of the amphibians from previous and recent surveys (black line). Red line shows the estimated number of unique, and duplicate species (blue line) of amphibians from long term surveys in Sekayu lowland forest, Hulu Terengganu.

From 14 species recorded in the first survey in 2003, the number of species had steadily increased to 18 species in 2008. The surveys continued in 2013 and recorded more species, with a total of 38 species in 2015. This trend of species discoveries kept increasing during the surveys up to the end of 2020. The results shown by the species accumulation curve generated from the list of species recorded between 2003 and 2018 show a constantly increasing trend (Fig. 12). The species accumulation curve has almost reached the asymptote, which indicates that our long-term surveys may have reached the true diversity of amphibians in SLF. The number of unique and duplicate species also kept decreasing over the years. However, even after our exhaustive fieldwork from 2015 to 2020, a few unique species such as *Phrynellapulchra*, *Rhacophoruspardalis*, *Thelodermahorridum*, and *Zhangixalustunkui* have remained unrecorded after their first discovery in 2013-2014. As for the records of amphibians in Hulu Terengganu District, this study contributes an additional 10 new records for this area, which now totals 70 species so far known (Table 2). The new records are Ichthyophiscf.asplenius, *K.palmatissimus*, *Kaloulalatidisca*, *Micrylettadissimulans*, *Microhylasuperciliaris*, *Nyctixaluspictus*, *Polypedatesdiscantus*, *Zhangixalusprominanus, Z.tunkui* and *Thelodermalicin*.

**Table 2.** Compilation of updated and revised checklist of amphibian fauna from published materials and this study in Hulu Terengganu, Terengganu. Note: DD = Data Deficient, NE = Not Evaluated, LC = Least Concerned, NT = Near Threatened, EN = Endangered, NP = Not Protected, and P = Protected. Symbol * = represents the additional record of species in Hulu Terengganu, 1 = Sekayu (This study), 2 = Gunung Lawit (Dring 1979; Sumarli et al. 2015), 3 = Tembat (Norhayati et al. 2011; Nur Amalina et al. 2017), and 4 = Gunung Gagau (Hamidi 2013).

No	Species	IUCN Status	WCA 2010	1	2	3	4
	**Family Ichthyophiidae**						
1	Ichthyophiscf.asplenius*	DD	NP	+	–	–	–
2	*Ichthyophisglutinosus*	VU	NP	–	–	+	–
3	*Ichthyophis* sp.	DD	NP	–	+	+	–
	**Family Bufonidae**						
4	*Ansonialatiffi*	NT	NP	+	+	–	+
5	*Ansonialumut*	NE	NP	+	+	–	–
6	*Duttaphrynusbengalensis* (*Duttaphrynus* sp.1)	LC	NP	+	–	+	–
7	*Ingerophrynusparvus*	LC	NP	+	+	+	+
8	*Ingerophrynusquadriporcatus*	LC	NP	–	+	+	–
9	*Leptophryneborbonica*	NE	NP	+	+	+	+
10	*Phrynoidisasper*	LC	NP	+	+	+	+
11	*Rentapiaflavomaculata*	NE	NP	+	+	+	–
	**Family Dicroglossidae**						
12	*Fejervaryacancrivora*	LC	NP	–	–	+	–
13	*Fejervaryalimnocharis*	LC	NP	+	+	+	+
14	*Ingeranatenasserimensis*	LC	NP	–	–	+	–
15	*Limnonectesblythii*	NT	P	+	+	+	+
16	*Limnonecteshascheanus*	LC	NP	+	–	–	+
17	*Limnonectesutara*	NE	NP	+	+	+	+
18	*Limnonectesdeinodon*	NE	NP	+	+	+	–
19	*Limnonectesmalesianus*	NT	P	+	–	+	–
20	*Limnonectestweediei*	LC	P	–	+	–	–
21	*Limnonectesparamacrodon*	NT	P	–	+	–	+
22	*Limnonectesplicatellus*	LC	NP	+	+	+	–
23	*Occidozygasumatrana*	LC	NP	+	+	+	–
24	*Occidozygamartensii*	LC	NP	+	+	–	–
	**Family Megophryidae**						
25	*Leptobrachiumhendricksoni*	LC	NP	+	+	+	+
26	*Leptobrachellaheteropus*	LC	NP	–	–	+	+
27	*Leptobrachellasola*	EN	NP	+	+	–	–
28	*Pelobatrachusnasutus*	LC	P	+	+	+	+
29	*Xenophrysaceras*	LC	P	–	+	–	–
	**Family Microhylidae**						
30	*Chaperinafusca*	LC	NP	–	–	+	–
31	*Kalophrynuskiewi*	NE	NP	+	+	+	–
32	Kalophrynuspalmatissimus*	EN	P	+	–	–	–
33	Kaloulalatidisca*	DD	NP	+	–	–	–
34	*Kaloulapulchra*	LC	NP	+	–	+	–
35	*Microhylabedmorei*	LC	NP	+	+	+	–
36	*Microhylabutleri*	LC	NP	+	+	+	–
37	*Microhylaheymonsi*	LC	NP	+	+	+	–
38	Microhylasuperciliaris*	LC	NP	+	–	–	–
39	*Microhylamantheyi*	LC	NP	–	+	–	–
40	Micrylettadissimulans*	LC	NP	+	–	–	–
41	*Metaphrynellapollicaris*	LC	NP	–	+	–	–
42	*Phrynellapulchra*	LC	NP	+	–	+	–
	**Family Ranidae**						
43	*Abavoranaluctuosa*	LC	NP	–	+	–	–
44	*Amolopsgerutu*	NE	NP	+	+	+	+
45	*Chalcoranalabialis*	NE	P	+	+	+	+
46	*Humeranamiopus*	LC	NP	+	–	+	–
47	*Hylaranaerythraea*	LC	P	+	–	+	+
48	*Indosylvirananicobariensis*	NE	NP	+	–	+	–
49	*Odorranahosii*	LC	P	+	+	+	+
50	*Pulchranabaramica*	LC	NP	–	–	+	–
51	*Pulchranaglandulosa*	LC	NP	+	–	+	–
52	*Pulchranalaterimaculata*	LC	NP	+	+	+	–
53	*Pulchranasundabarat*	LC	P	+	+	+	+
54	*Sylviranamalayana*	NE	NP	+	–	+	+
	**Family Rhacophoridae**						
55	*Kurixaluschaseni*	NE	NP	+	+	–	–
56	Nyctixaluspictus*	NT	P	+	–	–	–
57	*Philautusvermiculatus*	LC	NP	–	+	–	–
58	*Polypedatescolletti*	LC	P	+	+	+	–
59	Polypedatesdiscantus*	NE	NP	+	–	–	–
60	*Polypedatesleucomystax*	LC	NP	+	+	+	–
61	*Polypedatesmacrotis*	LC	NP	+	+	+	–
62	*Rhacophorusrhodopus*	LC	NP	–	+	–	–
63	*Rhacophorusnigropalmatus*	LC	P	+	+	–	–
64	*Rhacophoruspardalis*	LC	P	+	+	–	–
65	*Rhacophorusnorhayatiae*	NE	NP	–	+	–	–
66	*Thelodermaleprosum*	NE	NP	–	+	–	–
67	*Thelodermahorridum*	LC	NP	+	+	–	–
68	Thelodermalicin*	LC	NP	+	–	–	–
69	Zhangixalusprominanus*	LC	P	+	–	–	–
70	Zhangixalustunkui*	NE	NP	+	–	–	–
	Total numbers of species			**52**	**44**	**41**	**18**

## ﻿Discussion

This extensive survey on amphibian fauna in SLF highlights the immense biodiversity that can be found on a local scale through long-term inventory study, hence demonstrating the potential of Hulu Terengganu forests as one of the nation’s important biodiversity spots. The approach also affirms the importance of long-term and comprehensive surveys, regardless of substantial time, cost and effort are needed, especially in tropical countries. The long-term surveys in SLF have successfully captured the variation in abundance and composition of amphibians between habitats and the temporal patterns that are apparently impossible in short-term or rapid surveys. In addition, continuous, standardised sampling efforts and multi-habitat surveys could provide the most useful baseline information on the status and trends of amphibian communities in tropical lowland forest for biodiversity monitoring.

Amphibian community particularly in tropical forests are closely related to seasonal factors (Duellman and Trueb 1986; Praderio and Robinson 1990; Pearman et al. 1995), with many of the specialist or cryptic amphibians influenced by changing monsoon patterns (Gibbons and Bennett 1974; Bury and Corn 1987; Crosswhite et al. 1999). This can be indicated by the discovery of several new records during our recent surveys. For instance, seasonal breeding anurans such as *Micrylettadissimulans*, *Kaloulalatidisca*, and *Nyctixaluspictus*, (Chan et al. 2014b; Das et al. 2019), and rarer arboreal species like *Rhacophorusnigropalmatus* and *Thelodermalicin* were only found between October and early January. We can only assume they are influenced by the northeast monsoon (Muhammad et al. 2007; Mandeep 2008; Varikoden et al. 2011). Heavy rainfall would inundate and raise the temporary water bodies over the open forested areas and serve as a cue for breeding season for most of the tropical species (Rastogi et al. 2011).

Expanding the survey areas that have been overlooked before, such as hilly areas of forests and small streams, has been rewarding as additional species were added to the list. Hilly forested areas and small streams in fact serve as the better ground for foraging and breeding areas for many species of anurans (Pineda and Halffter 2004). As reported here, new records of anurans like *Leptophryneborbonica* and *Limnonectesplicatellus* were for the first time discovered under thick forest litter near the stream bank of the Peres River small stream, while *L.hascheanus* and *Thelodermalicin* were encountered for the first time at a long-abandoned hiking trail. We also started to realise that secretive and rarely found species like *Pelobatrachusnasutus*, *Rentapiaflavomaculata* and *Nyctixaluspictus* were actually abundant in pristine habitats such as the small stream of Peres River. After the continuous monthly surveys at this habitat, more specimens were obtained.

However, certain species such as *Phrynellapulchra*, *Thelodermahorridum*, and *Zhangixalustunkui* remained undetected since their initial discoveries in 2013–2014, even though continuous surveys have been made since 2015. This might indicate that these secretive species cannot be detected by a simple method such as visual surveys alone and may require alternative techniques that effective on arboreal amphibians. Several studies noted that *T.licin* and *P.pulchra* occupied smaller tree holes closer to the ground, generally in the less disturbed areas (McLeod and Norhayati 2007; Chan and Ahmad 2009). Thus, installing tree hole traps in non-recreational areas within SLF might be effective to sample these tree hole dwellers (Berry 1975; Duellman 1978; Yanoviak and Fincke 2005). Although *Zhangixalustunkui* shares the same habitat as its congener, which is rock pools along the upper stream areas of SRF, the species remains undetected, possibly because it is only present during the days with the heaviest rainfall in the monsoon season. Unfortunately, conducting fieldwork during such times is impossible due to limited visibility and the risk of fallen trees and wild animals.

The compilation of amphibian records from established inventories in Hulu Terengganu have demonstrated a great diversity of amphibians so far, with a total of 70 species compared to another studied area in Terengganu like Gunung Tebu and adjacent forests in Besut, which have a total of 50 amphibian species (Muin et al. 2014; Sumarli et al. 2015). The records of amphibians in Hulu Terengganu have surpassed the species richness reported from other localities with well-established inventories such as Endau-Rompin National Park (Kiew 1987; Lim 1989; Daicus and Hashim 2004; Norhayati and Shamada 2004; Wood et al. 2008a; Shahriza et al. 2012) and Krau Wildlife Park (Grandison 1972; Jasmi et al. 1999; Norsham et al. 2001; Chan et al. 2008; Zakaria et al. 2014). However, more areas in Hulu Terengganu remain unexplored, especially in Belukar Bukit and a large portion of Lake Kenyir catchment which surely holds vast unreported species diversity.

To date, there are 17 protected species of amphibians under the Wildlife Conservation Act 2010 (Act 716). Five species of ‘Near Threatened’, two species of ‘Endangered’ and one species of ‘Vulnerable’ amphibians found in Hulu Terengganu are listed on the IUCN Red List (IUCN, 2021). This area deserves attention for better protection of the species, especially for the endemic and threatened species in this area. More alarming, frequent changes in taxonomy and the description of many new species has put these species into a vulnerable state as most of them are not evaluated under the IUCN Red List. The problem with the Wildlife Conservation Act 2010 appears to be not comprehensive enough, and is outdated as there are only 17 species listed as protected, while many of the unique and newly described species such as *Rhacophorusnorhayatiae*, *Ansonialumut*, *Limnonectesutara*, and many others are not listed. This legislation requires immediate revision to offer better protection of these species from illegal trade and wildlife trafficking. Furthermore, many areas within Hulu Terengganu, even in SLF, are vulnerable to further deforestation if conservation action is not taken seriously. The benefits of this long-term data collection could be utilised to spread public awareness on the importance of biodiversity conservation and be extended for ecotourism benefits of this forest reserve.

## ﻿Conclusions

Fifty-two amphibian species from 32 genera were recorded from SLF, making up a total of 70 species recorded in Hulu Terengganu District. Data such as this are a clear indication that extensive study and monitoring is the cogent approach in attempting to reveal the true diversity of a forest reserve. Working repeatedly and systematically in this locality during different months of the years from 2003 to 2020 has resulted in revealing the ecological complexity and high species richness within this area. This finding also denotes that varying sampling efforts influence the knowledge on species diversity of the studied area. This study emphasised that continuous efforts of documenting species diversity is crucial to ensuring the reliability and validity of species diversity harboured by any area or habitat. The available information on amphibian diversity in SLF and Hulu Terengganu can hopefully be used to assist conservation programmes and long-term monitoring of biodiversity. In another way, remarkable species diversity of recreational forests and other areas should be preserved as it can be used to nurture conservation awareness and promote scientific citizenship amongst the local folks to work together to protect the biodiversity at this recreational forest.

## Supplementary Material

XML Treatment for Ichthyophis cf. asplenius

XML Treatment for Ansonia latiffi

XML Treatment for Ansonia lumut

XML Treatment for Duttaphrynus bengalensis

XML Treatment for Ingerophrynus parvus

XML Treatment for Leptophryne borbonica

XML Treatment for Phrynoidis asper

XML Treatment for Rentapia flavomaculata

XML Treatment for Fejervarya limnocharis

XML Treatment for Limnonectes blythii

XML Treatment for Limnonectes hascheanus

XML Treatment for Limnonectes deinodon

XML Treatment for Limnonectes malesianus

XML Treatment for Limnonectes plicatellus

XML Treatment for Limnonectes utara

XML Treatment for Occidozyga sumatrana

XML Treatment for Occidozyga martensii

XML Treatment for Kalophrynus kiewi

XML Treatment for Kalophrynus palmatissimus

XML Treatment for Kaloula latidisca

XML Treatment for Kaloula pulchra

XML Treatment for Microhyla berdmorei

XML Treatment for Microhyla butleri

XML Treatment for Microhyla cf. heymonsi

XML Treatment for Microhyla superciliaris

XML Treatment for Micryletta dissimulans

XML Treatment for Phrynella pulchra

XML Treatment for Leptobrachium hendricksoni

XML Treatment for Leptobrachella sola

XML Treatment for Pelobatrachus nasutus

XML Treatment for Amolops gerutu

XML Treatment for Chalcorana labialis

XML Treatment for Humerana miopus

XML Treatment for Hylarana erythraea

XML Treatment for Indosylvirana nicobariensis

XML Treatment for Odorrana hosii

XML Treatment for Pulchrana glandulosa

XML Treatment for Pulchrana laterimaculata

XML Treatment for Pulchrana sundabarat

XML Treatment for Sylvirana malayana

XML Treatment for Kurixalus chaseni

XML Treatment for Nyctixalus pictus

XML Treatment for Polypedates colletti

XML Treatment for Polypedates discantus

XML Treatment for Polypedates leucomystax

XML Treatment for Polypedates macrotis

XML Treatment for Rhacophorus nigropalmatus

XML Treatment for Rhacophorus pardalis

XML Treatment for Theloderma licin

XML Treatment for Theloderma horridum

XML Treatment for Zhangixalus prominanus

XML Treatment for Zhangixalus tunkui
